# Decoding cancer heterogeneity: studying patient-specific signaling signatures towards personalized cancer therapy

**DOI:** 10.7150/thno.31657

**Published:** 2019-07-09

**Authors:** Efrat Flashner-Abramson, Swetha Vasudevan, Ibukun Adesoji Adejumobi, Amir Sonnenblick, Nataly Kravchenko-Balasha

**Affiliations:** 1 Department for Bio-medical Research, Institute of Dental Sciences, Hebrew University of Jerusalem, Jerusalem 91120, Israel; 2 Oncology Division, Tel Aviv Sourasky Medical Center, and Sackler Faculty of Medicine, Tel Aviv University, Tel Aviv, Israel

**Keywords:** Personalized medicine, cancer combination therapy, information theory, protein networks, cell signaling, patient-specific network reorganization

## Abstract

The past years have witnessed a rapid increase in the amount of large-scale tumor datasets. The challenge has now become to find a way to obtain useful information from these masses of data that will allow to determine which combination of FDA-approved drugs is best suited to treat the specific tumor. Various statistical analyses are being developed to extract significant signals from cancer datasets. However, tumors are still being assigned to pre-defined categories (breast luminal A, triple negative, etc.), conceptually contradicting the vast heterogeneity that is known to exist among tumors, and likely overlooking unique tumors that must be addressed and treated individually. We present herein an approach based on information theory that, rather than searches for what makes a tumor similar to other tumors, addresses tumors individually and unbiasedly, and impartially decodes the critical patient-specific molecular network reorganization in every tumor.

**Methods**: Using a large dataset obtained from ~3500 tumors of 11 types we decipher the altered protein network structure in each tumor, namely the patient-specific signaling signature. Each signature can harbor several altered protein subnetworks. We suggest that simultaneous targeting of central proteins from every altered subnetwork is essential to efficiently disturb the altered signaling in each tumor. We experimentally validate our ability to dissect sample-specific signaling signatures and to rationally design personalized drug combinations.

**Results**: We unraveled a surprisingly simple order that underlies the extreme apparent complexity of tumor tissues, demonstrating that only 17 altered protein subnetworks characterize ~3500 tumors of 11 types. Each tumor was described by a specific subset of 1-4 subnetworks out of 17, i.e. a tumor-specific altered signaling signature. We show that the majority of tumor-specific signaling signatures are extremely rare, and are shared by only 5 tumors or less, supporting a personalized, comprehensive study of tumors in order to design the optimal combination therapy for every patient. We validate the results by confirming that the processes identified in the 11 original cancer types characterize patients harboring a different cancer type as well. We show experimentally, using different cancer cell lines, that the individualized combination therapies predicted by us achieved higher rates of killing than the clinically prescribed treatments.

**Conclusions**: We present a new strategy to deal with the inter-tumor heterogeneity and to break down the high complexity of cancer systems into simple, easy to crack, patient-specific signaling signatures that guide the rational design of personalized drug therapies.

## Introduction

A main feature of cancer cells is numerous oncogenic aberrations that disturb the balance of signaling networks, leading to abnormal integration and processing of external and internal cues in the cell 1,2. The cancer research community has come to realize that, in most cases, to treat cancer efficiently, a cocktail of drugs should be prescribed to patients, targeting several signaling pathways gone awry in the tumor cells. Some examples are combinations of EGFR and ERK inhibitors 3 and combinations of B-RAF and MEK inhibitors for melanoma 4. However, the extensive tumor heterogeneity poses a major obstacle. Tumors of the same type often respond differently to therapy, due to patient-specific molecular aberrations and/or untargeted tumor subpopulations 2. Although several studies have demonstrated a statistical ability to predict the response of a particular patient subgroup to a certain pre-selected treatment 5-7, we are still unable to predict *a priori*, which patient-specific combined therapy would be efficient 8.

To resolve inter-patient heterogeneity, a plethora of computational methods have been developed 9-11 such as reverse-engineering algorithms; multivariate statistical methods that include, for example, clustering methods and principal component analysis; Bayesian methods, based on elucidating the relationships between a few genes/proteins at a time 12; or machine learning 5,13. These methods frequently focus on characterizing the dominant, statistically significant, groups of co-varying molecules appearing in the entire patient population 11. However, these dominant groups may co-exist with additional, patient-specific groups, generating a unique structure of patient-specific altered networks. Furthermore, some of the patients may harbor rare altered network structures that do not include any of the dominant groups. Mapping patient-specific fold changes onto known pathways was recently used to reveal patient-specific rare alterations 14. However, similar oncogene/protein levels in different patients may stem from distinct molecular processes 15. This important information, which is necessary for drug design, may not be obtained by measuring fold changes. A researcher who plans to design a personalized drug combination must still rely on costly and time-consuming experimental screening of numerous drugs and available tissues from cancer patients 16-18.

Our aim is to step forward and develop a structured method that will describe individual tumors and patients in an accurate, unbiased manner, and enable prediction of potent patient-tailored drug cocktails, regardless of whether the specific tumor can be classified to a pre-defined group of tumors or not.

Tumor cells are influenced by different environmental and genetic constraints (e.g. crosstalk with stroma, genetic mutations, etc.). These constraints induce alterations in the protein network in the tumor. Importantly, not all altered proteins in a tumor are necessarily functionally connected to each other. Rather, they can be divided into subnetworks. These subnetworks may vary significantly between patients due to patient-specific mutations, i.e. each patient may harbor a different set of subnetworks, resulting in a patient-specific signaling signature. Furthermore, a certain protein can be altered to a similar extent in different patients but to participate in different subnetworks in those patients, due to patient-specific network reorganization 19.

We explore the data space of cancer patients utilizing an information-theoretic, thermodynamic-based method named surprisal analysis 20,21. The analysis unravels the constraints that operate in tumors and relates them to the experimentally-measured changes in protein expression levels. Our aim is to identify the unique structure of protein subnetworks that emerges, and accurately map every single tumor according to the molecular signaling signature that it contains.

The usage of a thermodynamic-based theory in biological systems is motivated by the ability to model the system and predict its behaviour in response to perturbation. Thermodynamic-based approaches 22-24 and information-theoretical approaches 25-27 have been successfully applied to the analysis of biological systems in a number of cases and have demonstrated predictive power.

We have successfully implemented surprisal analysis in a number of systems. For example, we demonstrated that the accurate identification of the signaling network structure that emerges in MCF10a human mammary cells upon stimulation with EGF, allowed us to anticipate the effect of the addition of protein inhibitors on the protein network structure 28. In other studies we have shown that surprisal analysis of cell-cell signaling in brain tumors provided a predicition about cellular spatial distributions and the direction of cell-cell movement 29,30.

Here we study a large proteomic dataset with a purpose to gain personalized information regarding the molecular processes underlying the inter-tumor heterogeneity among 3467 distinct tumors of 11 types, including breast, lung, brain and more. The dataset studied herein was generated and analyzed by Akbani et al., who showed that these 3467 tumors can be clustered into 8 groups, defined primarily by tumor type 31. Such a generalization of the data is bound to overlook specific tumors that differ from other tumors of the same type 31. The characterization we sought is such that every group of tumors contains tumors that are not only very similar*,* but are rather* identical* in terms of the set of altered protein subnetworks that they possess. We show herein that the majority of these groups each consist of only a *single* tumor, for which a unique therapy should be assigned.

We uncover the compilation of altered signaling subnetworks that characterizes this large and diverse collection of tumors, as well as the proteins that participate in each of these subnetworks. We show that only 17 subnetworks span the entire collection of tumors, appearing in different combinations of 1-4 subnetworks in each tumor. We focus on EGFR signaling to demonstrate the significant patient-specific reorganization of signaling subnetworks. We validate the results by verifying that the processes identified in the original 11 cancer types characterize patients harboring a different cancer type as well.

We end by analyzing a proteomic dataset obtained from 10 cancer cell lines and predicting drug combinations for each cell line. We demonstrate that deciphering the subset of altered subnetworks in every sample enables anticipating efficient targeted therapy combinations even for triple negative breast cancer, for which no targeted therapy has been approved yet. We further show that even though some cell lines are of the same genetic subtype, distinct drug combinations were predicted to be most efficient for each of the lines. Our predicted drug cocktails demonstrated exceptionally high potency, and achieved higher rates of killing than the clinically prescribed treatment.

## Results

### Surprisal analysis for the in-depth study of 3467 tumors of 11 different types

We performed surprisal analysis on a proteomic dataset that was obtained by subjecting samples from 3467 TCGA (The Cancer Genome Atlas 32) solid tumors to reverse phase protein array analysis 31 (**Table S1**). The tumors were of 11 different types: Breast (BRCA; n=747), colon adenocarcinoma (COAD; n=334), rectal adenocarcinoma (READ; n=130), kidney renal cell carcinoma (KIRC; n=454), ovarian cancer (OVCA; n=412), endometrial carcinoma (UCEC; n=404), lung adenocarcinoma (LUAD; n=237), head and neck squamous cell carcinoma (HNSC; n=212), lung squamous cell carcinoma (LUSC; n=195), bladder carcinoma (BLCA; n=127), and glioblastoma multiforme (GBM; n=215). The protein array included high-quality antibodies that target 181 proteins and phosphoproteins that play key roles in oncogenesis-related processes, such as proliferation, DNA damage, EMT, invasion, and apoptosis 31.

**Figure 1:**
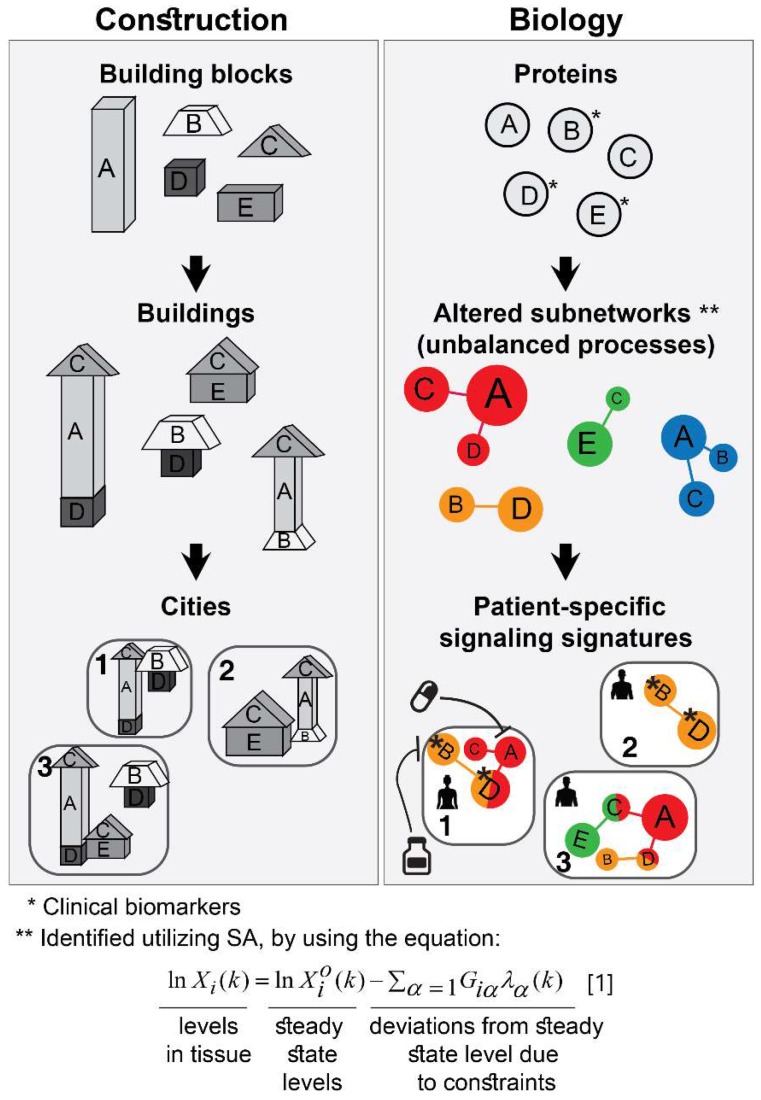
Overview of our approach. In construction, a collection of building blocks can be used to assemble different types of buildings (left). Specific subsets of buildings can exist in different cities (left). Similarly, in biology, altered protein subnetworks, or unbalanced molecular processes (buildings) are made up of a collection of proteins (blocks) (right). Each biological sample (city) can contain a unique subset of unbalanced processes that maintain its current state (right). This state is different from the steady state, since proteins participate in different unbalanced processes reflecting the genomic and environmental constraints on the tumor. Each protein can be influenced by several processes simultaneously (e.g. protein D in patient 1, right panel). In the figure, the proteins are represented by circles, such that the diameter of the circle denotes its relative weight (=importance), G, in the process (analogous to size of blocks in each building), e.g. protein A is important in the red process, less important in the blue process, and does not participate in the green and orange processes (right). The size of the entire process in each sample (or size of building in each city) denotes its sample-specific amplitude (λ). For example, the red unbalanced process is relatively important in patient 3 and insignificant in patient 2 (right). The common procedure today is to test biomarkers in cancer tissue (proteins B, D, E here). If B regulates D, the conventional approach would suggest that inhibition of B should inhibit D. While this inhibition may be effective in patient 2, it is expected to be inefficient in patient 1, because protein D participates in red and orange processes simultaneously in this patient. Refer to Supplementary Information for complete details regarding the mathematical algorithm.

The approach that we employ is comparable to construction. The first step consists of proteomic profiling for all tumors (**Figure 1**, right). This step is analogous to gathering a collection of building blocks (**Figure 1**, left). Next, surprisal analysis (SA) is utilized to decipher the altered subnetworks that appear in the entire population of tumors, and the proteins that take part in each subnetwork (**Figure 1**, right). This resembles the determination of the different types of buildings that can be built using the available collection of building blocks (**Figure 1**, left). Briefly, SA assumes that tumors are biological systems in which the balanced, steady state has been disturbed due to genomic and environmental factors, or constraints. Every constraint alters a part of the protein network structure in the tumor, such that a specific subnetwork of proteins undergoes coordinated changes in protein expression/activity levels. This subnetwork of co-varying proteins is defined as an *unbalanced process.* In other words, the unbalanced process is the subnetwork that was altered due to the constraint. SVD (Singular Value Decomposition 33,34) is used as a mathematical tool to fit the experimental data to the thermodynamic-based theory, represented by equation 1 (**Figure 1**, lower panel; refer to Supplementary Information (SI) for complete details).

Different subsets of buildings can appear in different cities (**Figure 1**, left). Similarly, different subsets of unbalanced processes are active in every patient (several constraints, and thus several processes, can operate on a given tumor at a given time; **Figure 1**, right).

The patient-specific subset of unbalanced processes, or altered subnetworks, provided by SA, constitutes the patient-specific altered signaling signature, and forms the basis for rational, personalized drug design. Importantly, SA provides information regarding the structure of the patient-specific signaling signature allowing to plan, *a priori*, how to collapse the entire unbalanced signaling flux in the specific tumor. To demonstrate this point, we consider patient 1 in Figure 1 (right panel, bottom). Should this patient be treated according to the traditional routine, his tumor would be examined histologically in search of potentially oncogenic biomarkers. For example, several protein biomarkers can be selected for measurement in a cancer type-specific manner (e.g. prostate-specific antigen (PSA) for prostate cancer, Her2, progesterone receptor, and estrogen receptor α (ERα) for breast cancer, etc.), and then the degree of upregulation/downregulation would be measured for each selected protein 35. Next, a treatment regimen would be chosen based on the targetable protein biomarkers discovered and the available FDA-approved drugs for the specific cancer type. In the example illustrated in Figure 1, patient 1 harbors two altered protein biomarkers, proteins B and D (**Figure 1**, right). Inhibition of protein B is expected to block signaling to protein D (according to known signaling pathways), and thus a drug against protein B can be selected for patient 1. However, SA revealed that this patient harbors two unbalanced processes, highlighted red and orange (**Figure 1**, right). Protein D is influenced by both processes (**Figure 1**, right). Therefore, inhibition of protein B alone may not suffice: unless the red process is also targeted (e.g. by targeting protein A), the activity of protein D is expected to persist in the tumor of patient 1 (**Figure 1**, right). We hypothesize that the complete set of unbalanced processes should be targeted simultaneously in order to effectively tackle the disease.

Importantly, our approach does not depend on prior knowledge regarding the cancer (type, anatomical origin) and is therefore unbiased.

To identify which processes are active in each tumor, SA assigns an amplitude, *λ_α_(k)*, for each process *α* in each patient *k* 15,21. If, in a certain patient, an unbalanced process is assigned an amplitude that exceeds the threshold limit (see SI, Methods), this process is considered active in the specific patient (the amplitude, *λ_α_(k)*, is analogous to the size of the building; **Figure 1**, bottom left). To reveal which proteins are involved in each process, each protein is assigned a weight, ***G_iα_***, reflecting the extent of participation of each protein in each process. If, in a certain process, a protein is assigned a weight that exceeds the threshold limit (see SI, Methods), this protein is considered to be influenced by the specific process (the weight, *G_iα_* , is analogous to the size of the block in a specific building; **Figure 1**, middle left). A detailed explanation regarding the analysis can be found in the SI.

### 17 unbalanced processes construct 3467 patient-specific signaling signatures

**Figure 2:**
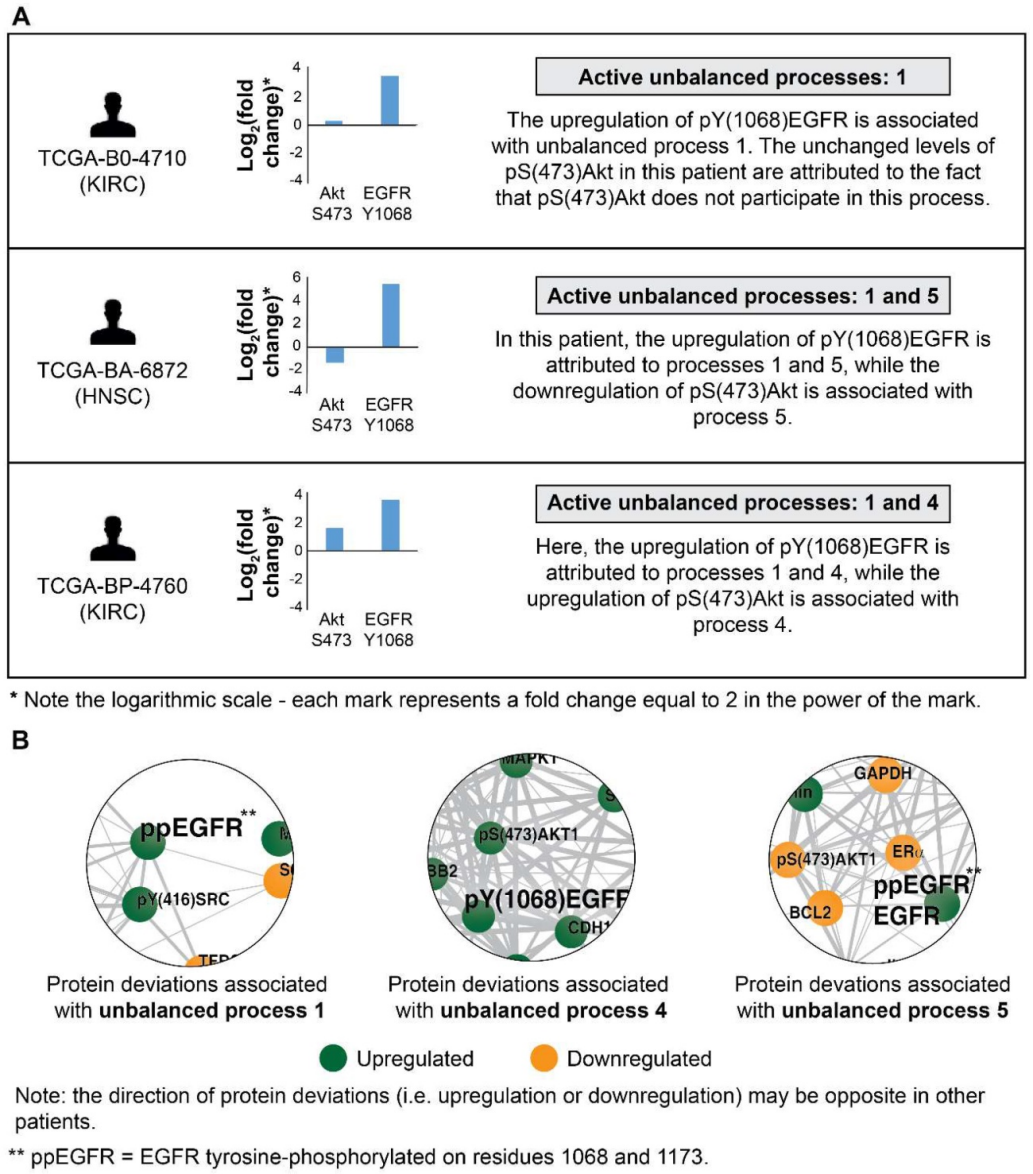
EGFR signaling undergoes significant changes in different patients. (A) 3 representative patients were chosen in order to demonstrate the significant reorganization of the EGFR signaling pathway in each patient. All patients harbor increased pY(1068)EGFR (middle panel). This upregulation is associated with different subsets of unbalanced processes (right panel). (B) Zoom in images of the protein deviations associated with unbalanced processes 1, 4, and 5 are shown. To determine the direction of change in every protein (i.e. upregulation or downregulation) the amplitudes of the processes in these patients were considered. Note that in other patients the directions of change may be opposite. See SI for more details. The complete subnetworks are presented in Figure S1.

Here, we uncovered the unbalanced processes that appear in the entire dataset of 3467 tumors (**Table S1**), and then assigned a patient-specific subset of processes to each and every tumor. We found that only 17 unbalanced processes characterize the entire population of 3467 tumors (**Table S2, Figure S1**). Not all processes were active in all patients. Rather, each patient was characterized by a specific subset of 1-4 unbalanced processes out of 17 (**Figure 2**, **Table S3**). See SI and Figure S2 for a discussion of how the 17 processes were determined, and how they suffice to reproduce the experimental data.

The dataset included tumor samples only, and therefore the unbalanced processes identified by SA characterize the inter-tumor heterogeneity. 17 groups of co-varying proteins, constructed from the array of 181 proteins and phosphoproteins tested, are enough to describe the biological alterations that differentiate 3467 tumors. Considering the size of the dataset and the variety of tumors it contains, this result is highly interesting. Each tumor is characterized by a specific subset of unbalanced processes, and therefore 17 unbalanced processes “allow” a very high degree of inter-tumor heterogeneity. Thousands of distinct combinatorial possibilities for sets of 1-4 unbalanced processes can be chosen out of 17. On the other hand, the fact that each tumor can be portrayed by a small subset of unbalanced processes unmasks a surprisingly simple order that underlies the very large complexity of cancer systems.

The proteins participating in the different unbalanced processes were assembled into subnetworks, in which pairs of co-varying molecules were connected using functional interactions according to STRING database 36 (**Figure 2B**, **Figure S1**). Visualization of the protein-protein connections in each process allows to select druggable targets. Proteins marked red in Figure S1 exhibited a change in expression level that is opposite from that of proteins marked blue. Note that these colors indicate only the correlation or anti-correlation between the proteins. The actual direction of change (i.e. upregulation or downregulation; as indicated in **Figure 2B**) can be deciphered based on the sign of the amplitude of the process (see SI for a detailed explanation). The process of determination of the amplitudes for every unbalanced process is described in SI Methods. Figure S3 demonstrates how we determined which proteins participate in every unbalanced process.

The variety of signaling signatures that appear in the different tumors is what underlies the disparities in protein expression levels between different patients (**Figure 2**, **Table S3**). Note that each unbalanced process may include several signaling pathways, which co-vary in a coordinated manner, e.g. one pathway can be upregulated and the other downregulated, both can be upregulated together, etc. (**Figure S1**, **Table S2**). This is an important attribute of SA, because it simplifies the design of therapy for every patient: while a specific tumor may demonstrate aberrations in multiple signaling pathways, these pathways may change in a coordinate manner and thus be represented by fewer unbalanced processes. We hypothesize that targeting one or two central hub proteins from each unbalanced process will be enough to reduce the patient-specific unbalanced signaling flux.

To verify the robustness and accuracy of the analysis we randomly picked 100 patients from each type of cancer (generating a training dataset - 1100 patient total, representing about a third of the complete dataset), and found that the patient characterization remained essentially the same (**Figure S4**).

### Surprisal analysis provides patient-specific altered signaling signatures

The ability to decipher patient-specific signaling signatures in an accurate manner is crucial to the design of patient-tailored medicine. To demonstrate how SA addresses this issue, we inspected EGFR signaling in 3 patients (**Figure 2**). In patient TCGA-B0-4710, proteomic profiling revealed that pY(1068)EGFR was upregulated while one of its major downstream signaling proteins, pS(473)Akt, remained unchanged (**Figure 2A**, top). The analysis shows that this patient harbors only unbalanced process 1, in which pY(1068)EGFR participates, but pS(473)Akt does not. Thus, the upregulation of pY(1068)EGFR and the unchanged levels of pS(473)Akt are attributed to this process (**Figure 2B** and **Figure S1A**). Interestingly, this decoupling between EGFR and Akt occurs in unbalanced process 1, which is the *most significant* unbalanced process, being active in 48.3% of the whole population of 3467 tumors (such decoupling events have been reported previously, see 37).

Patient TCGA-BA-6872 demonstrated an increase in pY(1068)EGFR, and a decrease in pS(473)Akt (**Figure 2A**, middle). The signaling signature of this patient consists of two unbalanced processes, 1 and 5 (**Figure 2A**, middle). The upregulation of pY(1068)EGFR is attributed to both unbalanced processes, 1 and 5, while the decrease in pS(473)Akt is associated with process 5 (**Figure 2B** and **Figure S1A,E**). EGFR and Akt are anti-correlated in the unbalanced processes 7 (**Figure S1G**), 10 (**Figure S1J**), and 14 (**Figure S1N**) as well, appearing in a total of 24.6% of the 3467 tumors.

Patient TCGA-BP-4760 exhibited an upregulation in both pY(1068)EGFR and pS(473)Akt (**Figure 2A**, bottom). SA revealed that this patient harbors processes 1 and 4 (**Figure 2A**, bottom). Upregulation of pY(1068)EGFR is attributed to both processes, while the upregulation pS(473)Akt is associated with process 4 (**Figure 2B** and **Figure S1A,D**). Thus, in this patient inhibition of pS(473)Akt, for example, will not necessarily reduce the entire signaling flux, since process 1 is not expected to be influenced 28. EGFR and Akt are *correlated* in the unbalanced process 13 as well (**Figure S1M**; appearing in 16.6% of the tumors).

Another major downstream effector of pY(1068)EGFR is pT(202)Y(204)MAPK. Indeed, our analysis revealed that pY(1068)EGFR and pT(202)Y(204)MAPK are *correlated* in the unbalanced processes 1 and 14 (**Figure S1A,N**; appearing in 49.3% of the tumors). However, they are *anti-correlated* in the unbalanced processes 7, 10 and 13 (**Figure S1G,J,M**; appearing in 12.2% of the tumors), and *non-correlated* (pT(202)Y(204)MAPK is absent) in the unbalanced processes 4 and 5 (**Figure S1D,E**; appearing in 15.5% of the tumors).

### Utilizing the complete sets of patient-specific unbalanced processes is essential for the efficient mapping of 3467 patients

We wished to utilize the comprehensive data obtained by SA for the development of a simple method to design patient-specific combination therapies. To this end, we sought to achieve efficient mapping of the 3467 patients.

Figure S5 shows the amplitudes of the 17 unbalanced processes for all patients of each cancer type. The graphs demonstrate that there is some degree of cancer type commonality. For example, the majority of GBM tumors display a signature comprising a subset of the unbalanced processes 1, 2, 3, 5, 6 (**Figure S5A**). However, when looking at the *complete set* of tumor-specific unbalanced processes it becomes evident that the majority of GBM tumors are not alike. For example, of the 215 GBM patients, 202 patients were found to harbor unbalanced process 2, while only 7 of the GBM tumors harbor unbalanced process 11, and only 4 tumors harbor unbalanced process 12 (**Table S2**). This notion is especially important when devising anti-cancer treatments: we hypothesize that the *entire* tumor-specific unbalanced network needs to be targeted to effectively treat the tumor. Knowledge about what is common among tumors may not be enough. In other words, attempting to assign cancer patients to pre-existing groups of patients based on molecular *commonalities* may overlook important *differences* between the tumors and significantly impact treatment efficacy and success.

Next, we investigated patient-specific signaling signatures of processes. To study the recurrence of the different signaling signatures (each comprising a different set of unbalanced processes) in the different tumors, we defined a specific *barcode* for each individual tumor, representing the tumor-specific signaling signature. Namely, the amplitudes of the unbalanced processes were normalized such that they equal one of three values: -1, 0, or 1 (see SI). This way the entire collection of tumor-specific signaling signatures can be compared to one another. The list of barcodes and their recurrence in the different patients can be found in Table S4. We found that 452 distinct barcodes repeated themselves in the 3467 tumors. 17 unbalanced processes can, in principle, give rise to many thousands of different tumors, not only 452. Therefore, it seems that despite the massive inter-tumor heterogeneity it is plausible to classify cancer patients into groups.

**Figure 3:**
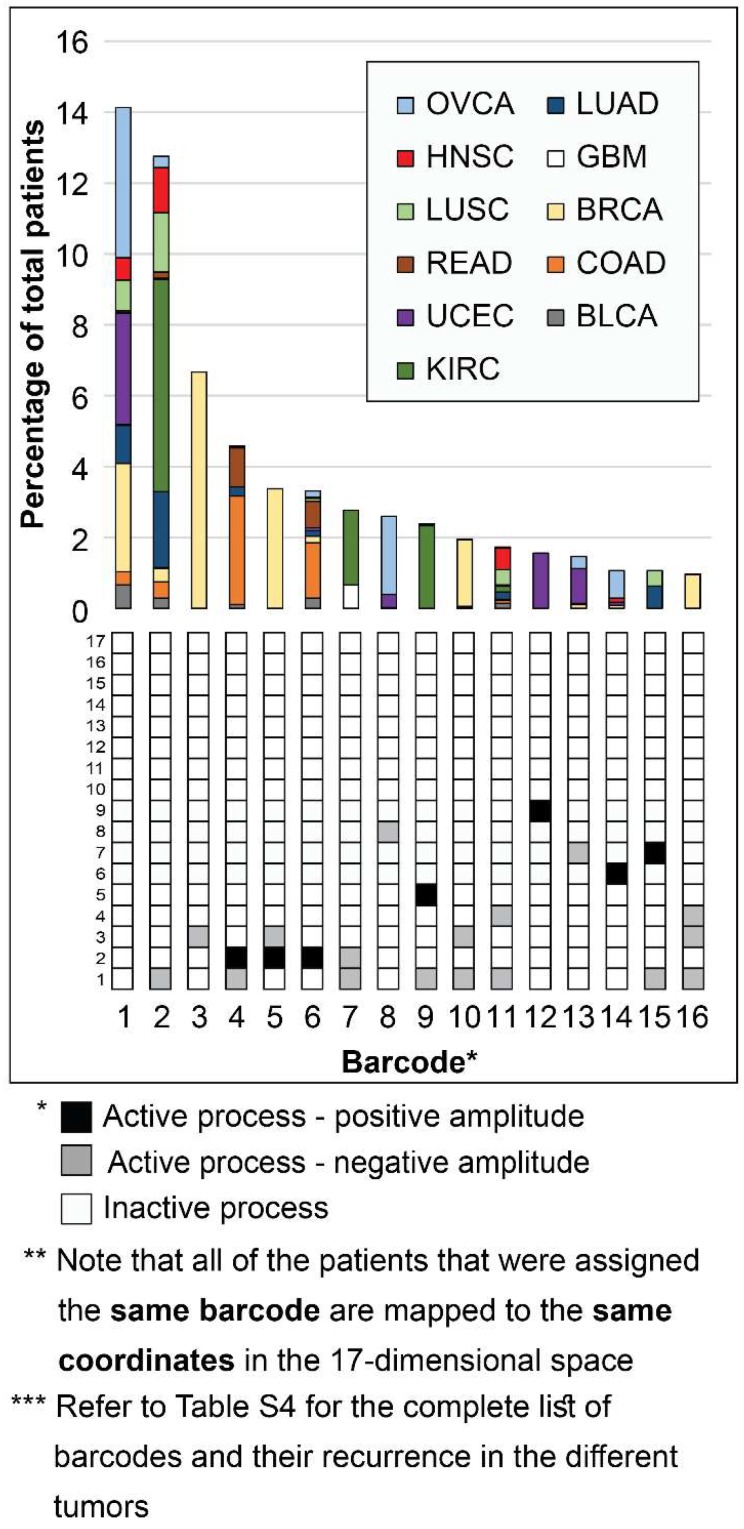
Mapping the patients into a 17-dimensional data space. Mapping the patients into a data space, where each dimension is represented by a specific unbalanced process, enables to accurately describe every individual tumor. The 16 most abundant barcodes are shown, each appearing in 1% or more of the patients (at least 35 patients). Each tumor-specific barcode we identified is mapped to specific coordinates in the 17-dimensional space, according to the active unbalanced processes and the sign of their amplitude. The graph presents a two-dimensional form of this concept - each column in the graph represents multiple patients that were mapped to the same coordinates in the 17-dimensional space. The complete information derived using surprisal analysis of the 3467 tumors can be mapped using 452 distinct barcodes (see Table S4), which are mapped to 452 different coordinates in the 17-dimensional space. Note: Active unbalanced processes are such that were assigned a significant amplitude (normalized to 1 or -1, marked black or gray, respectively), whereas inactive unbalanced processes are such that were assigned an insignificant amplitude (normalized to 0 and marked white).

Interestingly, while 16 barcodes were relatively abundant (i.e. each represent 1% or more of the population of tumors), most barcodes were extremely rare: 376 barcodes (indexed 77-452 in **Table S4**) each represent only 5 tumors or less. 273 of these rare barcodes (indexed 180-452 in **Table S4**) each represent only a *single* patient (see also **Figure S6A**). To assign the correct therapy to these patients, it is vital to inspect each of the tumors individually and accurately. Existing methods usually classify cancer patients by generating 2D maps using a limited number of components, thereby potentially overlooking important patient-specific molecular information (exemplified here in **Figure S7**). In contrast, the representation of tumors according to the barcode of unbalanced processes that they possess enables to map each and every patient (**Figure 3**). Transformation of the proteomic data obtained from the tumors into a 17-dimensional space, represented by the 17 distinct unbalanced processes identified, allows to map every single patient into this space, including patients that harbor one-of-a-kind tumors. We demonstrate this in Figure 3 for the 16 most abundant barcodes (each representing at least 35 patients (1%), indexed 1-16 in **Table S4**). Each column in the graph represents a specific barcode of unbalanced processes, color-coded according to the different cancer types that possess this barcode. Once the data is represented this way, a wealth of information can be extracted. For example, it is evident that most of the barcodes represent multiple cancer types, i.e. most bars contain multiple colors. Additionally, each cancer type is represented by multiple barcodes, i.e. each color appears in multiple bars in the graph.

Notably, the most abundant barcode (indexed 1 in **Figure 3** and **Table S4**, and appearing in 14.1% of tumors) is the null barcode, containing no unbalanced processes (**Figure 3**). This means that tumors harboring this barcode are non-heterogeneous in the pathways captured by the current array of proteins. The representation of the data as shown in Figure 3 allows to gain important insights into this finding. For example, none of the GBM tumors in the dataset were assigned the null barcode, suggesting that the protein array tested provides sufficient coverage for the molecular processes that are heterogeneously altered in GBM malignancies. The unbalanced processes in the KIRC tumors in the dataset were also fully covered by the array. On the contrary, 35.7% of OVCA patients and 27% of UCEC patients were assigned the null barcode, suggesting that these tumors do not differentially express the proteins tested (**Figure 3** and **Table S4**; Elaborated on in the Discussion).

Note that most GBM patients are *not* represented in the graph in Figure 3. While ~10% of GBM patients are represented by barcode 7 (**Figure 3**), most GBM patients are each represented by a rare barcode. This finding is true for all tumor types - out of the collection of barcodes that represent the specific population of tumors, the vast majority of barcodes are rare, i.e. appear only in 5 tumors or less. 47 of the 51 barcodes in BLCA tumors are rare (**Figure S6B**). 54 of the 65 barcodes that represent GBM tumors are rare (**Figure S6C**). 40 of the 46 barcodes in LUSC are rare (**Figure S6D**). In general, at least 78% of the barcodes were rare in each of the tumor type tested (**Figure S6**).

Another interesting finding is related to BRCA tumors. Barcodes 3, 5, and 10 represent almost exclusively BRCA patients (**Figure 3**). These barcodes contain the unbalanced processes 1, 2 and 3, that include oncogenes such as estrogen receptor α (ERα), androgen receptor (AR), EGFR, VEGFR2, and more (**Figure S1** and **Table S2**). However, *not all BRCA patients are represented by these three barcodes*. To map all BRCA patients, the complete set of unbalanced processes should be examined. For example, there is a single BRCA patient that harbors barcode 66, containing the unbalanced processes 1, 4, and 7 (**Table S4**). Another one-of-a-kind BRCA patient harbors barcode 73, containing the unbalanced processes 2 and 7 (**Table S4**). Many more BRCA patients may be overlooked unless their tumors are all analyzed in terms of the complete array of 17 unbalanced processes.

Genomic analysis is routinely used in clinics, in order to determine the pathological state of tumors and to assign therapy to the patients. We randomly checked pairs of patients with the same proteomic phenotype, i.e. the same barcode, in this dataset (e.g. patients 2109 and 2328; 1526 and 3140; 2604 and 2650 in**Table S3**) and based on the lists of hot-spots 38 (oncogenic or likely oncogenic mutations) found that their genomic profiles differ significantly (**Table S3;** according to cBioPortal database). Therefore, the genetic information obtained in clinics may not suffice to design personalized drug cocktails.

### Surprisal analysis efficiently predicts anti-cancer combination therapies

To provide a proof of concept that the signaling signatures identified by SA can be utilized to rationally design efficacious personalized drug cocktails, we turned to analyze a cell line proteomic dataset, which can be experimentally validated in the laboratory.

**Figure 4:**
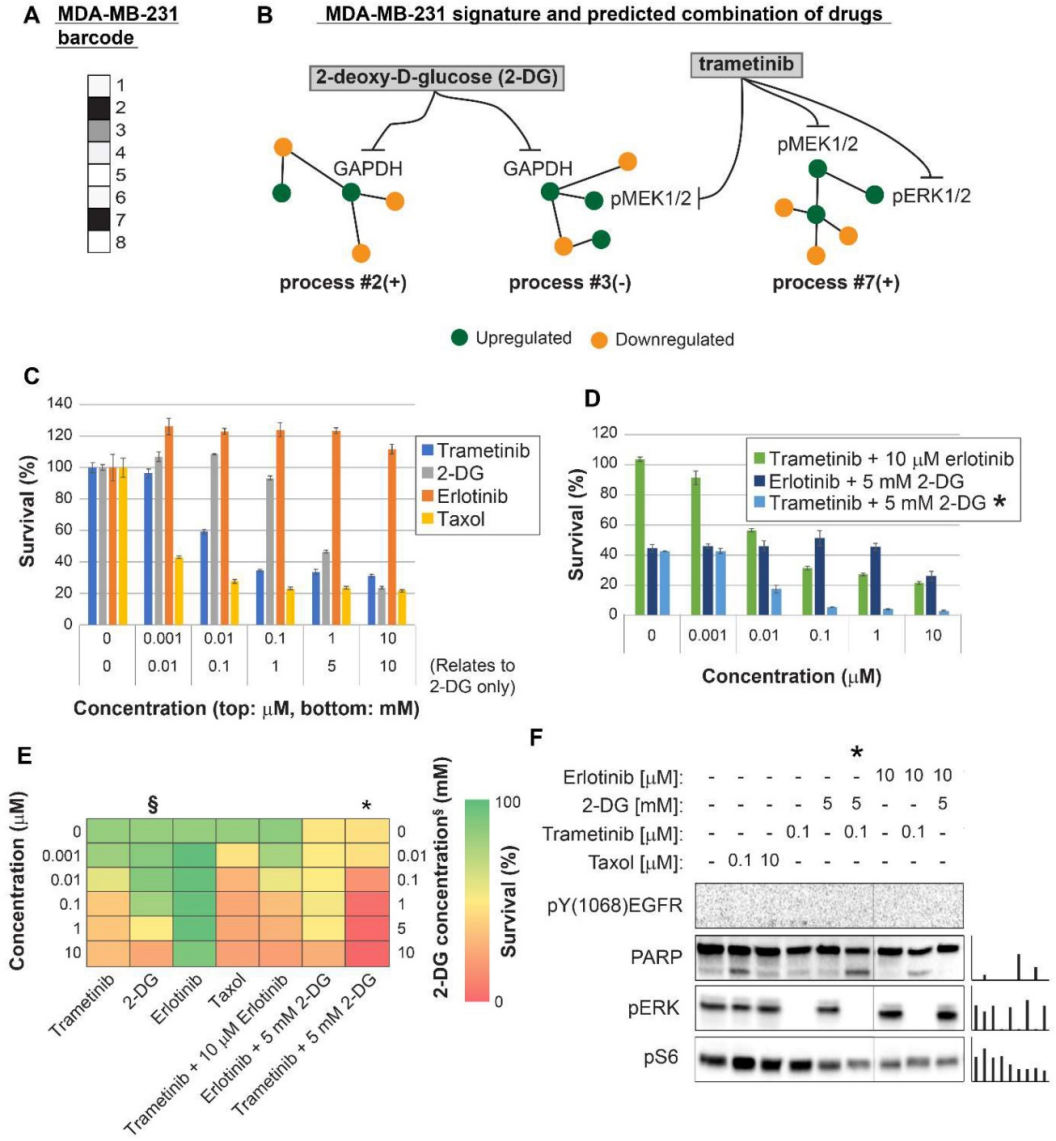
Surprisal analysis predicts efficient drug combinations for MDA-MB-231 TNBC cell line. (A,B) The barcode of unbalanced processes and the emerging altered signaling signature of MDA-MB-231 cells, according to SA. Schematic figures of each unbalanced process are shown, including the sign of the amplitude in the specific cell line ((-) or (+)). Accordingly, the upregulation or downregulation of every protein is indicated in green or yellow, respectively. Note that in specific samples the directions of change may be opposite, e.g. the proteins in process 7 undergo opposite deviations in MDA-MB-231 and MDA-MB-468 vs. MCF7 (see Figures 5 and 6, panels B). See SI for more details. The complete subnetworks are presented in Figure S8. Drug prediction is indicated, including the processes each drug is predicted to target. (C,D,E) Survival rates in response to different treatments. The combination of drugs predicted to target the complete unbalanced signaling signature was tested (marked with an asterix), as well as combinations that were predicted to only partially target the unbalanced signaling flux, each drug alone and taxol. The results are also shown in heatmap form for improved clarity. (F) Western blot analysis of the cells following different treatments. Our predicted drug combination induced high levels of PARP cleavage relative to other treatments. All results were normalized to total lane intensity obtained by stain-free imaging (Bio-Rad). The column graphs present the normalized results.

**Figure 5:**
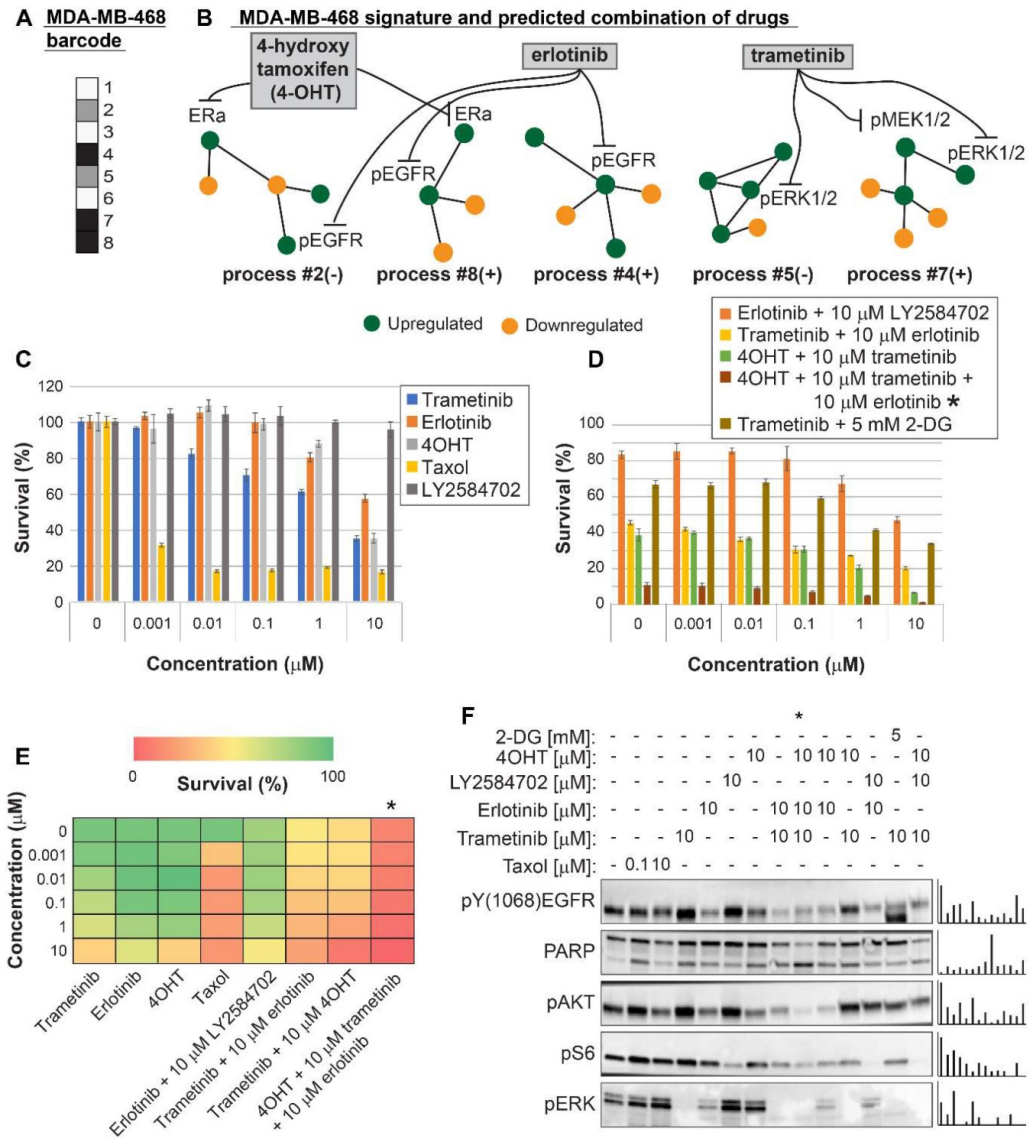
Surprisal analysis predicts efficient drug combinations for MDA-MB-468 TNBC cell line. (A,B) The barcode of unbalanced processes and the emerging altered signaling signature of MDA-MB-468 cells, according to SA. Schematic figures of each unbalanced process are shown, including the sign of the amplitude in the specific cell line ((-) or (+)). Accordingly, the upregulation or downregulation of every protein is indicated in green or yellow, respectively. Note that in specific samples the directions of change may be opposite, see SI for more details. The complete subnetworks are presented in Figure S8. Drug combination prediction is indicated. (C,D,E) Survival rates in response to different treatments. The combination of drugs predicted to target the complete unbalanced signaling signature was tested (marked with an asterix), as well as combinations that were predicted to only partially target the unbalanced signaling flux, each drug alone and taxol. The combination predicted to target MDA-MB-231 was tested (trametinib + 2-DG), showing significantly lower efficacy against MDA-MB-468 cells. The results are also shown in heatmap form for improved clarity. (F) Western blot analysis of the cells following different treatments. Our predicted drug combination induced high levels of PARP cleavage relative to other treatments. All results were normalized to total lane intensity obtained by stain-free imaging (Bio-Rad). The column graphs present the normalized results.

**Figure 6:**
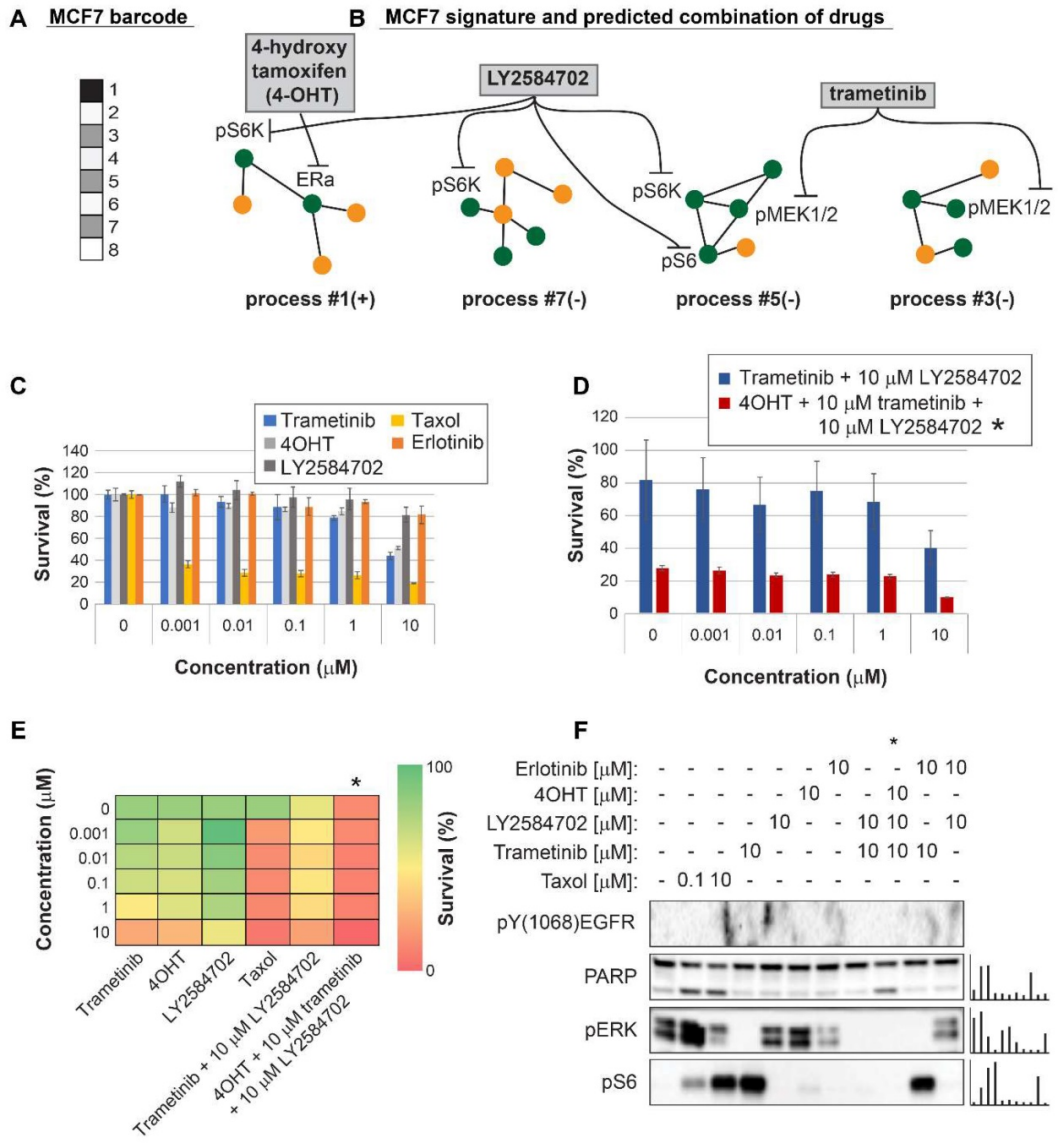
Surprisal analysis predicts efficient drug combinations for MCF7 luminal type A cell line. (A,B) The barcode of unbalanced processes and the emerging altered signaling signature of MCF7 cells, according to SA. Schematic figures of each unbalanced process are shown, including the sign of the amplitude in the specific cell line ((-) or (+)). Accordingly, the upregulation or downregulation of every protein is indicated in green or yellow, respectively. Note that in specific samples the directions of change may be opposite, see SI for more details. The complete subnetworks are presented in Figure S8. Drug combination prediction is indicated. (C,D,E) Survival rates in response to different treatments. The combination of drugs predicted to target the complete unbalanced signaling signature was tested (marked with an asterix), as well as combinations that were predicted to only partially target the unbalanced signaling flux, each drug alone and taxol. The results are also shown in heatmap form for improved clarity. (F) Western blot analysis of the cells following different treatments. Our predicted drug combination induced high levels of PARP cleavage relative to other treatments. All results were normalized to total lane intensity obtained by stain-free imaging (Bio-Rad). The column graphs present the normalized results.

The cell line dataset was obtained from 10 cancer cell lines, including breast, ovarian, and esophageal cancer 39. The dataset was analyzed using SA (**Table S5**, **Figure S8**) and then drug combinations were predicted for each cell line according to the specific altered signaling signature that it harbors (**Figures 4-6, panels A,B**, and **Table S6**). The predictions were made such that for each cell line at least one protein hub from every active unbalanced process will be inhibited.

Three breast cancer cell lines were randomly chosen for experimental validation: MDA-MB-231 (**Figure 4**), MDA-MB-468 (**Figure 5**), and MCF7 (**Figure 6**). The first two represent triple negative breast cancer (TNBC) against which no targeted therapy exists in clinics today. TNBC is often treated with non-specific chemotherapy, such as taxol 40. MCF7 represent luminal type A breast cancer, routinely treated with the ERα inhibitor, tamoxifen, and in some cases chemotherapy such as taxanes 41.

MDA-MB-231 cells were indeed efficiently killed by taxol treatment (**Figure 4C,E**). However, the efficacy of the treatment plateaued at 100 nM (**Figure 4C,E**). The MEK1/2 inhibitor, trametinib, was predicted to be partially effective, because the MAPK pathway participated in the imbalance in these cells (**Figure 4A,B, Table S6**, **Figure S8**). Trametinib, however, was not expected to collapse the entire imbalance in the cells, and indeed a plateau was reached at 100 nM (**Figure 4C,E**). We predicted that since MDA-MB-231 cells harbor two distinct unbalanced processes, both processes should be targeted to efficiently kill the cells (**Figure 4B**). To this end, we combined trametinib with the glycolysis inhibitor, 2-deoxy-D-glucose (2-DG) (**Figure 4B,D,E, Table S6**, **Figure S8**). Indeed, the combination was found to be exceptionally effective, and more efficacious than each inhibitor alone or taxol (**Figure 4D,E**). The combination also brought about reduced signaling in both processes, as indicated by the representative targets and the enhanced cleavage of the apoptotic marker PARP (**Figure 4F**). A lower intensity of cleaved PARP was observed when each inhibitor was applied alone (**Figure 4F**). In contrast, the EGFR inhibitor, erlotinib, showed no effect on the survival of these cells, alone or when combined with trametinib (**Figure 4C,D,E**). Moreover, erlotinib inhibition alone did not influence the levels of its canonical target, pERK (**Figure 4F**). This is because EGFR was not found to participate in the signaling imbalance in these cells (when cultured under complete medium; see **Tables S5** and **S6**). Hence, ERK is induced but not regulated by EGFR, as predicted by the analysis. These results demonstrate that the analysis correctly identified the atypical organization of the EGFR network in this cell line.

For MDA-MB-468 cells we initially predicted a double combination consisting of trametinib and erlotinib, since these drugs should target all 5 unbalanced processes that were found to be active in MDA-MB-468 cells (**Figure 5A,B**, **Table S6**, **Figure S8**). The combination killed up to ~75% of the cells, comparable to the rate of killing achieved by taxol (~80%), and more effective than each drug alone (**Figure 5C,D,E**). The combination treatment also induced PARP cleavage (**Figure 5F**). We then postulated that since the signaling signature of MDA-MB-468 cells consisted of 5 unbalanced processes (**Figure 5A,B**), and since processes 2, 4 and 8 had at least 2-fold higher amplitudes than in other cell lines (**Table S5**), these cells may require an additional drug that will enhance the inhibition of the imbalance. Therefore, we tested the survival of the cells when treated with a triple combination in which the ERα inhibitor, 4-hydroxy tamoxifen (4OHT; the activated derivative of tamoxifen) was added to trametinib and erlotinib. 4OHT was predicted to target processes 2 and 8, thereby supporting the actions of erlotinib in these cells (**Figure 5B**). Indeed, the triple combination induced near complete killing of MDA-MB-468 cells (**Figure 5D,E**) and depletion of the cellular signaling (**Figure 5F**).

We tested the combination predicted for MDA-MB-231 cells, trametinib and 2-DG, on MDA-MB-468 cells showing that it was much less effective in the killing of MDA-MB-468 cells, as predicted by our analysis (**Figure 5D**). Figure S9 shows the cross-check comparison of the effects of the predicted combinations on both TNBC cell lines. Our results demonstrate that cancer cell lines of the same type may require a different combination treatment, and that our approach resolves the optimal combined treatment for each malignancy.

For MCF7 cells (Luminal A subtype) we predicted that using 4OHT alone (as would be suggested for these cells based on the ERα biomarker) would only partially inhibit the unbalanced signaling flux in these cells, targeting one out of four unbalanced processes that are active in these cells (**Figure 6A,B**). Indeed, 4OHT killed only up to 50% of the cells (**Figure 6C,E**). We demonstrate that 4OHT does not reduce the entire signaling, by showing no effect on the pERK pathway, as expected by our analysis** (Figure 6F)**. We predicted that to collapse the entire imbalance in these cells, a triple combination, consisting of trametinib, LY2484702 (an inhibitor of p70S6K), and 4OHT would be more effective (**Figure 6A,B**, **Table S6**, **Figure S8**). The triple combination was indeed highly effective, surpassing each inhibitor alone, taxol, or the combination of trametinib and LY4584702 without 4OHT (**Figure 6C,D,E**). Similar to the case in MDA-MB-468 cells, the double combination consisting of trametinib and LY2584702 was expected to target all unbalanced processes in these cells. However, since there are 4 distinct unbalanced processes that need to be targeted, addition of a third drug was necessary to enhance the effect (**Figure 6A,B**). These results coincide with the results obtained in western blot, showing that the triple combination achieved the most significant PARP cleavage and the depletion of signaling in the cells (**Figure 6F**). This result provides an additional example of how the clinically prescribed therapy can be significantly improved by rationally designing drug combinations based on knowledge of the specific altered signaling signature.

### Suggesting patient-specific combination therapies

**Figure 7:**
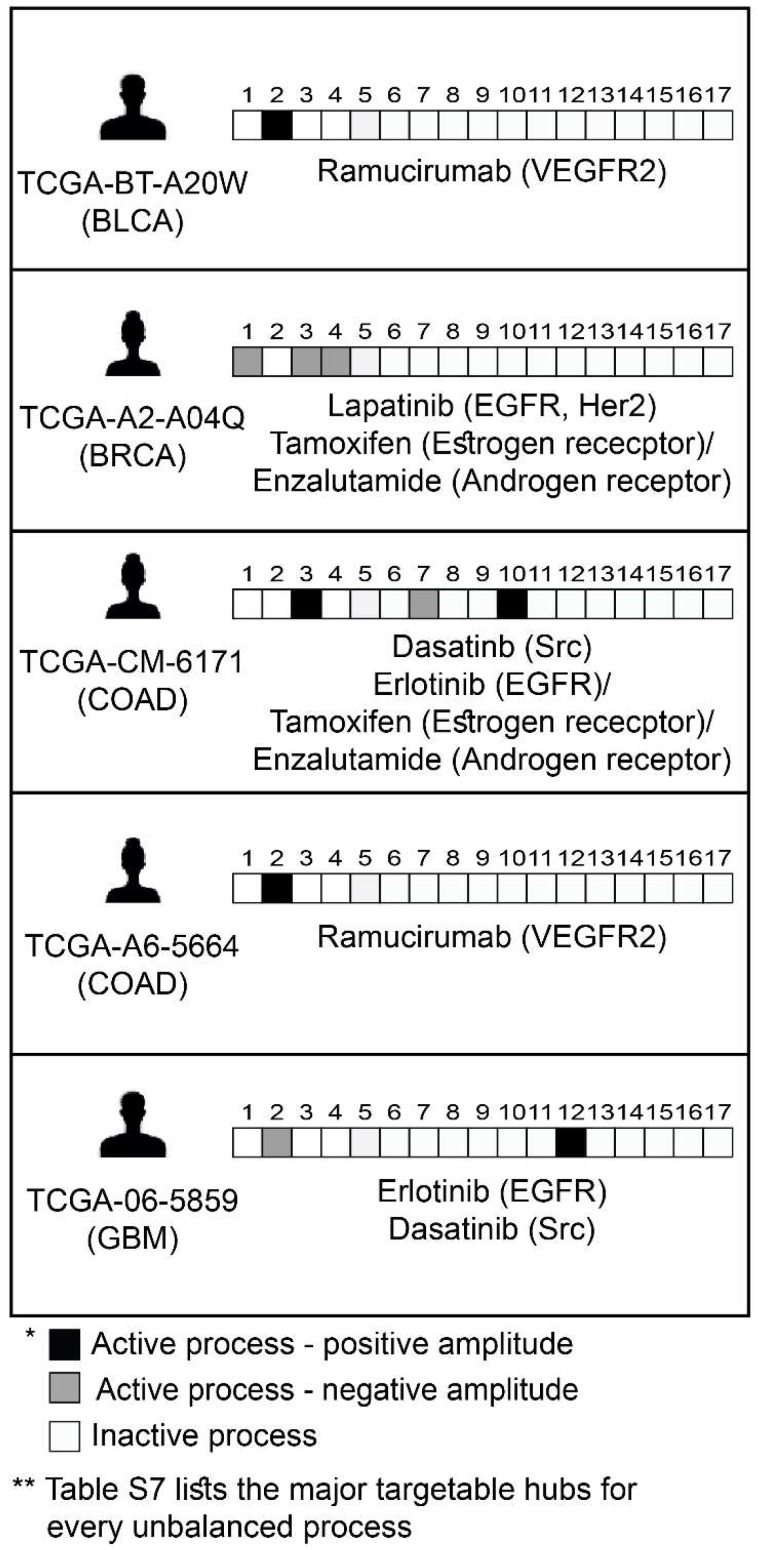
Prediction of patient-specific combination therapy. Five random patients were chosen, and their tumor-specific barcodes were inspected. Next, their tumor-specific unbalanced networks were examined, and the major targetable hubs were chosen. In the final step, a combination therapy that should target the entire unbalanced signaling flux in each tumor was suggested, consisting of FDA-approved drugs against the major hubs. (The list of FDA-approved drugs assigned to the different unbalanced processes can be found in Table S7. The complete list of suggested patient-specific combination therapies can be found in Table S8).

Encouraged by our experimental results, we returned to the large dataset of 3467 tumor, to suggest personalized combinations of drugs predicted to reduce the inter-tumor heterogeneity. Each unbalanced process was examined individually, the major targetable hubs were chosen, and FDA-approved drugs were assigned accordingly to each process 42 (**Table S7**). Then, each patient was assigned a combination of drugs according to the signaling signature that his tumor harbors. Figure 7 exemplifies this process for 5 patients, and the complete list of proposed patient-specific drug combinations can be found in Table S8.

Note that for each process several alternative protein targets can be suggested (**Table S7**). As long as the entire set of unbalanced processes is targeted, the specific drug targets can be selected by a researcher/clinician based on practical considerations, such as inhibitor availability, drug costs, overall toxicity, and drug interactions in the combined treatment.

Various drug combinations were suggested for different tumors of the same type (**Table S8**). For example, until today only one targeted therapy was approved for the treatment of BLCA (Atezolizumab, an antibody against programmed cell death-ligand 1 (PD-L1)). Our analysis suggests that BLCA patients can be treated with combinations that in many cases include erlotinib (an EGFR inhibitor, approved for the treatment of lung and pancreatic cancer), ramucirumab (an antibody against VEGFR2, approved for the treatment of colon cancer, adenocarcinoma of the stomach and lung cancer) and tamoxifen (an ERα inhibitor, approved for the treatment of breast cancer).

Another example is HNSC: Targeted therapies for the treatment of HNSC include anti-PD-1/PD-L1 and anti-EGFR antibodies. Our analysis indeed suggested combination therapies encompassing erlotinib for the majority of HNSC patients. However, the suggested combinations for the HNSC patients in the dataset also frequently include FDA-approved drugs such as tamoxifen and dasatinib (a Src/Abl dual inhibitor, approved for the treatment of chronic myelogeneous leukemia and acute lymphoblastic leukemia).

In clinics, ER-positive (ER+) BRCA patients would be suggested an ER-targeting drug (e.g tamoxifen; alone or in combination with other drugs). We predicted that 43% of the 469 ER+ patients in the dataset would indeed benefit from the monotherapy (**Figure S10**). However, the rest of the ER+ patients were suggested combinations of drugs including tamoxifen and other targeted therapies, none of which are currently used to treat BRCA patients in clinics. Examples include tamoxifen (or another ER inhibitor) combined with erlotinib (against EGFR), and tamoxifen combined with ramucirumab (against VEGFR2) (**Figure S10**). These combinations were indeed proposed by recent studies as potential effective combinations for ER resistant breast cancer (**Figure S10**) 43,44.

Drug combinations predicted for additional cancer types are also supported by the experimental findings of others. Some examples can be found in Figure S10. These results support and strengthen our findings.

### The processes identified in 11 cancer types fully characterize an independent dataset of patients harboring another tumor type

**Figure 8:**
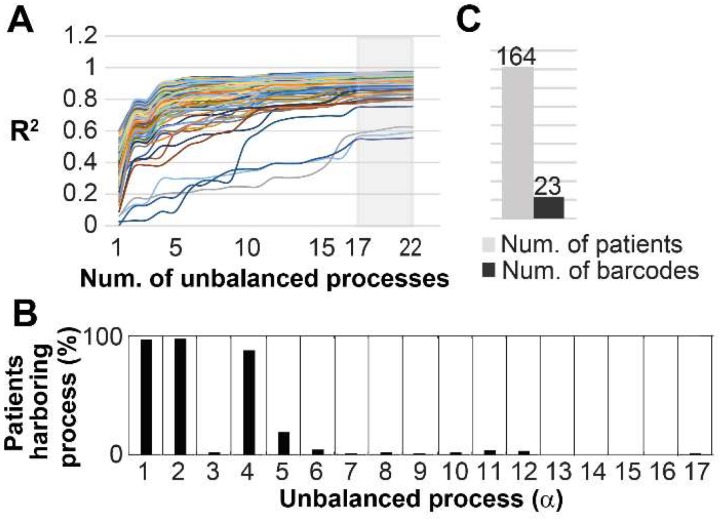
The 17 unbalanced processes fully characterize 164 prostate adenocarcinoma patients. (A) R2 values were calculated for all PRAD patients by plotting the natural logarithm of the experimental data ln(Xi(k)) against ΣGiαλα(k) (the sum of the protein level alterations due to unbalanced processes, SI and 15) for different values of α. The value of R2 approaches a plateau as more unbalanced processes are added to the calculation. Mathematically, 181 unbalanced processes are calculated for each patient. However, not all of them are significant. The figure shows that the plots for all patients reach a plateau after 17 processes, suggesting that the 17 unbalanced processes are significant, and the rest of the processes represent random noise in the tissues. 3 patients had especially noisy protein profiling as their R2 plots reached the plateau at the value 0.6 (B) The frequency of the unbalanced processes in 164 patients. (C) 23 barcodes, representing distinct altered signaling signatures, were identified for 164 PRAD patients.

Finally, we asked whether our findings are relevant in a broader sense, namely can the 17 unbalanced processes identified in the original 3467 patients describe additional cancer patients. To this end we examined an independent dataset from the TCPA portal 39 that included 164 patients harboring prostate adenocarcinoma (PRAD). The data from these patients was added to the original dataset, and the combined data was analyzed utilizing SA. Figure 8A shows that all 164 PRAD patients were fully described by the 17 unbalanced processes identified for the original 3467 patients, as convergence of the experimental data with the calculated unbalanced processes was reached after 17 processes. Figure S11 shows that the protein composition of the processes did not change as a result of adding the PRAD dataset. The most dominant unbalanced processes in PRAD patients were processes 1, 2 and 4 (**Figure 8B**). However, additional less dominant processes were found in this subgroup of patients, generating 23 barcodes, or 23 distinct altered signaling signatures (**Figure 8C**, **Table S9**). 20 of the PRAD barcodes are rare, characterizing only 5 PRAD patients or less, of them 16 representing *single* PRAD tumors (**Figure 8C**, **Table S9**).

### A proposed approach for personalized cancer therapy

**Figure 9:**
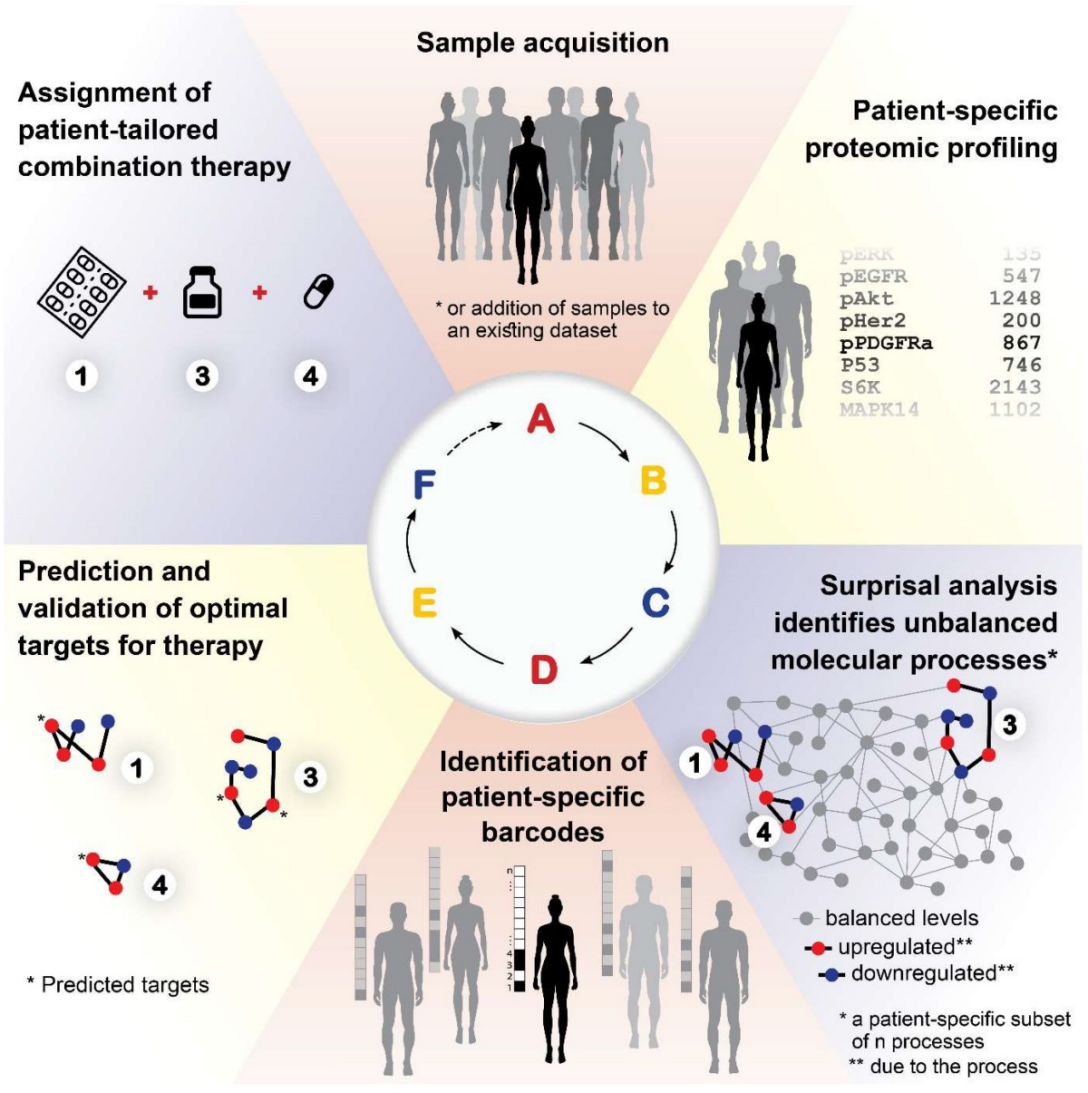
A proposed approach for personalized cancer therapy. Following acquisition of samples (A), a dataset is constructed using proteomics techniques (B). Surprisal analysis is then utilized (C) in order to uncover the complete patient-specific protein network structure, comprising balanced and unbalanced molecular processes, in which all molecules undergo coordinated changes in expression. Next, a patient-specific barcode is constructed, indicating the set of significant unbalanced processes that influence the specific tumor (D), and the tumor-specific unbalanced network is examined, aiming to identify and verify experimentally the major hubs whose blockage will lead to a collapse of the unbalanced network (E). Finally, a tumor-specific combination of targeted therapies is tailored to every patient (F).

Based on our results, we propose the following approach for the development of a personalized cancer therapy regimen: Following acquisition of tumor samples (**Figure 9A**), proteomic profiling will be performed for all samples (**Figure 9B**). The data will be analyzed computationally utilizing SA and compared to matching non-cancer control samples, to identify the unbalanced molecular processes that have emerged in each and every tumor (**Figure 9C**). Once the patient-specific barcodes have been elucidated (**Figure 9D**), the optimal drug targets will be predicted and tested experimentally (**Figure 9E**), and then a patient-tailored combination therapy will be assigned to the patients (**Figure 9F**). Once we obtain a large enough database, additional patients/samples can be added to the analysis individually, without the need to collect multiple samples at once.

## Discussion

In this study we aimed to find a way to accurately map cancer patients according to their tumor-specific molecular aberrations. For this purpose, we implemented an information-theoretic approach to the study of a large cohort of 3467 tumors of 11 different types. The first and most striking result we obtained is that 17 unbalanced processes describe the differential expression of 181 cancer-related signaling proteins in 3467 tumors. Each individual tumor harbors a specific barcode, comprising a subset of a few unbalanced processes, which describes the protein network reorganization in the specific patient. This result is highly significant from two aspects: (1) It is in line with the high degree of inter-tumor heterogeneity that is known to exist among tumors. 17 unbalanced processes can be assembled into thousands of unique barcodes comprising 1-4 active processes; (2) Our findings suggest that the cohort of patients consists of 452 types of cancer, rather than only 11 types. These 452 types, each representing a signaling signature, or a combination of 17 unbalanced processes, are mapped into a low-dimensional space, consisting of 17 dimensions. In our recent transcriptomic analysis of ~550 patients, in which every patient was profiled for over 20,000 different transcripts, we achieved a similar compaction of the heterogeneous molecular data 15, showing that ~550 different tumors could be fully characterized by 13 gene expression unbalanced subnetworks.

We show that the patient-specific signaling signature assigned to each patient allows designing patient-tailored combinations of drugs, many of which already exist in clinics. Moreover, some of the combinations predicted by SA for specific cancer types are supported by experimental findings of others.

We demonstrate the generality of our findings by adding an independent dataset of patients with prostate cancer. We found that the original 17 unbalanced processes could fully characterize this new group of prostate cancer patients.

It is of note that larger coverage of the proteome may allow resolving the complete set of unbalanced processes with higher precision. This can be achieved simply by enlarging the RPPA chip to contain additional signaling pathways. In case that a larger coverage of the proteome will identify a larger number of unbalanced processes, the number of dimensions in the data space into which the proteomic data is transformed should merely be increased. The proposed procedure for designing patient-specific medicine remains essentially the same.

Nonetheless, the dataset analyzed here comprised 181 proteins and phosphoproteins, including many well-known hubs in tumor-associated signaling networks. Interestingly, Wei at el. showed recently that an analysis of only 12 proteins on the single cell level enabled resolving alterations in signaling networks in response to therapy, while an in-depth genomic analysis failed to discover such changes 45.

We presented herein experimental results, showing that combination therapies predicted for cancer cell lines by the SA-based analysis proved to be highly efficacious. TNBC is the most lethal among breast cancer subtypes 40. Until today, no targeted therapy has been approved for TNBC, and therefore TNBC patients are currently treated with non-specific chemotherapy. Importantly, tumors that are classified as TNBC do not necessarily share much beyond triple-negativity, namely lack of expression of ERα and progesterone receptor (PR), and lack of overexpression of Her2, and thus represent a molecularly heterogeneous group of tumors 40. We show herein that using SA, we were able to capture the molecular differences between TNBC MDA-MB-231 and MDA-MB-468 cell lines. These differences give rise to distinct signaling signatures. Consequently, different combinations of targeted therapies were predicted to be efficacious for each of these cell lines. Our experimental results show that our predictions for these TNBC lines were highly successful. Additionally, we show that the luminal type A MCF7 cells can benefit from the addition of two targeted drugs to the clinically used tamoxifen, corresponding to the results we obtained in the analysis of the large patient dataset where we showed that the majority of ER+ breast cancer patients should benefit from combining tamoxifen with other targeted therapies.

Studies are underway in our laboratory, aiming to optimize therapy predictions, considering the size of the unbalanced processes, the optimal type of drug, etc. With the continued development of efficient tools for the generation of functional proteomics data of biological samples, our approach can eventually maturate into a valuable approach that can be used in research and clinics to produce therapeutic predictions in a small amount of time. We believe that our approach will provide the means to crack the patient-specific structures of protein networks, and advance the field of cancer therapeutics towards truly personalized medicine.

## Materials and Methods

**Datasets.** The large dataset of 3467 human tumors was obtained from the TCGA database 32. Each of the samples in this dataset was measured once (**Table S1**). The human cell line dataset used for experimental validation was obtained from the TCPA portal 39. In this dataset each cell line was grown under various conditions, e.g. following growth under complete media, under starvation, or following treatment with metformin or 2-DG, summing up to 6 conditions for each cell lines and 60 samples in total (**Table S5**). The measurement of multiple samples for each cell line serves to increase the resolution of our analysis.

**Surprisal analysis.** The analysis was carried out as described before 15,21. A detailed explanation regarding the thermodynamic considerations, the mathematical algorithm and implementation of the analysis in this study can be found in the text and SI.

Determination of the number of significant unbalanced processes. As described previously 15,28. See also SI.

**Generation of functional subnetworks.** The functional subnetworks presented in Figure S1 were generated using a python script (written with the assistance of Mr. Jonathan Abramson). The goal was to generate a functional network according to STRING database, where proteins with negative G values are marked blue and proteins with positive G values are marked red, to easily identify the correlations and anti-correlations between the proteins in the network. See SI for further information.

**Calculation of barcodes.** The barcodes presented in Figures 4-7, Tables S4, S8 and S9, and in Figure S6 were generated using a python script (written with the assistance of Mr. Jonathan Abramson), which normalized the patient-specific significant amplitude values to -1, 0 and 1. See SI for further details.

**Cell lines and reagents.** MDA-MB-231 (triple negative breast cancer), MDA-MB-468 (triple negative breast cancer), and MCF7 (lumA breast cancer) were obtained from the American Type Culture Collection (ATCC) and cultured in RPMI with 10% fetal calf serum (FCS), supplemented with 100 U/ml penicillin and 100 mg/ml streptomycin, and grown at 37ºC/5% CO2. The cell lines were authenticated at the Biomedical Core Facility of the Technion, Haifa, Israel.

For more details and reagents, see SI.

**Methylene blue assays.** Cells were treated as indicated for 72 hours and then survival was quantified by methylene blue staining, as described previously 46. Also elaborated in SI.

**Western blot analysis.** Cells were treated as indicated for 48 hours, and then adherent and floating dead cells were collected, lysed, and analyzed by western blotting as described previously 46. Also elaborated on in SI. All results were normalized according to total lane intensity, which was obtained using the stain-free imaging technology (Bio-Rad). Normalized results are presented in column graphs to the right of the blot images in Figures 4-6.

## Supplementary Material

Supplementary methods and figures.

Table S1.

Table S2.

Table S3.

Table S4.

Table S5.

Table S6.

Table S7.

Table S8.

Table S9.

## Abbreviations

SA: surprisal analysis SA
TNBC: triple negative breast cancer
EGFR: Epidermal growth factor receptor
pEGFR: phosphorylated EGFR
ERK: extracellular signal-regulated kinase
MAPK: mitogen-activated protein kinase
MEK: mitogen-activated protein kinase
FDA: Food and Drug Administration
ER: estrogen receptor
PR: progesterone receptor
HER2: human epidermal growth factor receptor-2
SVD: Singular Value Decomposition, the linear algebra procedure used to diagonalizable a matrix
G: Weight for each protein, reflecting the extent of participation of each protein in the unbalanced process an
*λα*: amplitude, an extent of activity of an unbalanced process in each patient
Akt: Protein kinase B
pAkt: phosphorylated Akt
MB: Methylene blue assay
PARP: Poly (ADP-ribose) polymerase
MDA-MB-231: triple negative breast cancer (TNBC) cell line
MDA-MB-468: triple negative breast cancer (TNBC)
MCF7: luminal type A breast cancer cell line
4OHT: the activated derivative of tamoxifen
2-DG: 2-deoxy-D-glucose
LY: LY2484702, an inhibitor of p70S6K
trametinib: MEK inhibitor
erlotinib: EGFR inhibitor
