# Supplementary material for: Decoding cancer heterogeneity: studying patient-specific signaling signatures towards personalized cancer therapy
Source: Theranostics. 2019 Jul 9;9(18):5149–65. doi: 10.7150/thno.31657 (PMC6691586; doi:10.7150/thno.31657)
Supplement: Supplementary file 1 — Supplementary methods and figures. [file thnov09p5149s1.pdf]

Supplementary Information

Decoding cancer heterogeneity: studying patient-specific signaling signatures towards personalized cancer therapy

Efrat Flashner-Abramson[1#], Swetha Vasudevan[1#], Ibukun Adesoji Adejumobi[1], Amir Sonnenblick[2] and Nataly Kravchenko-Balasha[1,*]

[1]Department for Bio-medical Research, Institute of Dental Sciences, Hebrew University of Jerusalem, Jerusalem 91120, Israel

[2] Oncology Division, Tel Aviv Sourasky Medical Center, and Sackler Faculty of Medicine, Tel Aviv University, Tel Aviv, Israel

*Corresponding author: natalyk@ekmd.huji.ac.il

[#] Equal contribution

Table of content:

Supplementary Methods 3

Surprisal analysis. 3

Determination of the number of significant unbalanced processes. 6

Generation of functional subnetworks. 7

Calculation of barcodes. 7

Cell lines and reagents. 8

Methylene blue assays. 8

Western blot analysis. 8

Supplementary Figures and legends 9

Figure S1. 9

Figure S2. 12

Figure S3. 13

Figure S4. 14

Figure S5. 15

Figure S6. 16

Figure S7. 18

Figure S8. 19

Figure S9. 20

Figure S10. 21

Figure S11. 22

References 23

Supplementary Methods

Surprisal analysis.

Surprisal analysis is a thermodynamic-based information-theoretic approach [1–3]. The analysis is based on the premise that biological systems reach a balanced state when the system is free of constraints [4–6]. However, when under the influence of environmental and genomic constraints, the system is prevented from reaching the state of minimal free energy, and instead reaches a state which is higher in free energy (in biological systems, which are normally under constant temperature and constant pressure, minimal free energy equals maximal entropy).

For example, if the system under study is a living cell, an environmental constraint can be exposure to a drug, which inflicts a change in protein concentrations and activities in the cell. The system can be influenced by genomic constraints as well, such as genomic mutations that in turn affect protein function, often eliciting alteration of specific signaling pathways to oppose the functions of the damaged protein.

Surprisal analysis can take as input the expression levels of various macromolecules, e.g. genes, transcripts, or proteins. However, be it environmental or genomic alterations, it is the proteins that constitute the functional output in living systems, therefore we base our analysis on proteomic data. The varying forces, or constraints, that act upon living cells ultimately manifest as alterations in the cellular protein network. Each constraint induces a change in a specific part of the protein network in the cells. The subnetwork that is altered due to the specific constraint is termed an unbalanced process. System can be influenced by several constraints thus leading to the emergence of several unbalanced processes. When tumor systems are characterized, the specific set of unbalanced processes is what constitutes the tumor-specific signaling signature.

For every protein, *i*, surprisal analysis calculates its expected expression level when the system is at the steady state and free of constraints: X_i^0. This term was shown to be constant, i.e. is independent of time and of the actual state of the system [7–10]. In terms of information theory, X_i^0 represents the state of maximal entropy, or minimal information.

$X_i(k)$ is the actual, experimentally measured expression level of the protein *i* in the tumor *k*. In cases where $X_i(k) \neq X_i^0$, we assume that the expression level of the protein *i* was altered due to constraints that operate on the system. Surprisal

analysis discovers the complete set of constraints operating on the system in any given tumor, k, by utilizing the following equation [7]:

$$\ln X_i(k) = \ln X_i^0(k) - \Sigma G_{i\alpha}\lambda_\alpha(k) \quad \textbf{(1)}$$

The term $\Sigma G_{i\alpha}\lambda_\alpha(k)$ represents the sum of deviations in expression level of the protein i due to the various constraints, or unbalanced processes, that exist in the tumor k. The processes are indexed α =1, 2, 3, … , such that the dominance of the process decreases with increasing index, i.e. unbalanced process 1 is active in more tumors than unbalanced processes 2, 3 etc.

The difference between the steady state expression level, X_i^0, and the actual expression level, $X_i(k)$, represents the amount of information we have about protein i. A protein that is influenced by constraints, i.e. is influenced by one or more unbalanced processes and is therefore functionally linked to other proteins, cannot take any possible expression level. Rather, its expression level is affected by the expression levels of other proteins.

The term $G_{i\alpha}$ denotes the degree of participation of the protein i in the unbalanced process α, and its sign indicates the correlation or anti-correlation between proteins in the same process. For example, in a certain process α, proteins can be assigned the values: $G_{\text{protein1},\alpha} = -0.50$, $G_{\text{protein2},\alpha} = 0.24$, and $G_{\text{protein3},\alpha} = 0.00$, indicating that this process altered proteins 1 and 2 in opposite directions (i.e. protein 1 is upregulated and protein 2 is downregulated, or vice versa due to the process α), while not affecting protein 3. Note that each protein can take part in a number of unbalanced processes at once.

Importantly, not all processes are active in all tumors. The term $\lambda_\alpha(k)$ represents the importance of the unbalanced process α in the tumor k. Its sign indicates the correlation or anti-correlation between the same processes in different tumors. For example, if the process α is assigned the values: $\lambda_\alpha(1) = 3.1$, $\lambda_\alpha(20) = 0.0$, and $\lambda_\alpha(138) = 2.5$, it means that this process influences the tumors of the patients indexed 1 and 138 in the same direction, while it is inactive in patient 20.

The partial deviations in expression level of the protein i due to the different constraints sum up to the total change in expression level (relative to the balance state level), $\Sigma G_{i\alpha}\lambda_\alpha(k)$.

Surprisal analysis uses a covariance matrix of the surprisals, meaning the covariance of the natural logarithms, as an *intermediate numerical* step (*11).* This has a physical, rather than purely statistical, significance [7]. That matrix has a

mathematical form dictated by the theory, which relates changes in protein concentrations to changes in chemical potentials (see mathematical explanation below). A change in chemical potential is directly related to the change in the free energy of the system. Therefore, changes in chemical potential of molecules allow us to predict the direction of biochemical reactions, and the behavior of the system [11]. Hence, our method allows interpretation of protein expression level changes from a different angle, i.e. according to thermodynamic-based laws. We have recently demonstrated that the approach allowed us to predict the direction of cell-cell movement [12,13], or the response to drugs [14]. Furthermore we have recently compared surprisal analysis to the pure statistical techniques widely used in biology, such as Principle component analysis and clustering methods, and have found that surprisal analysis provided a higher degree of resolution of biological cancer heterogeneity [9]. The detailed explanation about the main differences between surprisal analysis and other techniques can be found in [15].

A change in chemical potential for each protein is calculated using $\ln X_i(k) = \ln X_i^0(k) + (\mu_i - \mu_i^0)/kT$. This equation is developed further, to obtain the main equation in the text: (1) $\ln X_i(k) = \ln X_i^0(k) - \Sigma G_{i\alpha}\lambda_\alpha(k)$. Thus, a change in the chemical potential of protein i, $(\mu_i - \mu_i^0)/kT$, is represented by $\Sigma G_{i\alpha}\lambda_\alpha(k)$, denoting that the change in chemical potential occurred due to genomic and environmental constraints that operate in the tumor, indexed $\alpha = 1, 2, \ldots$ [3,12]. The method of Lagrange undetermined multipliers is utilized to calculate the maximal entropy and to determine the $\lambda_\alpha(k)$ values.

To fit the sum of the terms, as shown on the right-hand side of the main equation ([1]), to the logarithm of the measured expression level of protein i in a tumor k we use singular value decomposition (SVD). SVD serves as a method for diagonalizing the matrix that includes the logarithm of protein intensities in tumor k. In other words, SVD is used as a fitting method to bridge between the theory: $\ln X_i(k) = \ln X_i^0(k) - \Sigma G_{i\alpha}\lambda_\alpha(k)$; and experimental protein expression levels [7].

We determine the minimal number of unbalanced processes needed to accurately reconstruct the experimental protein expression levels, as described in the next section. For more details regarding the mathematical analysis see references [7] and [12]. For comparison between surprisal analysis and common statistical methods used today, such as Principle component analysis and clustering methods see [15,16].

As explained above, the zeroth term, $\ln X_i^0(k)$, is the expression level at the steady state of the system (the most significant process), invariant term, which was found not to change between patients or in time [7–10]. This term is

utilized as a reference against which the deviation terms are identified. In the dataset analyzed in this manuscript, the expression levels of the proteins were normalized according to the median values. Since steady state distribution is composed of these reference values (ln $X_i^0(k)$), mean/median centering of the data does not allow to determine this distribution explicitly. The zeroth term becomes a vector of zeros for all proteins *i* in all samples, and the dataset is fitted to the unbalanced processes. Equation (1) is reduced to the form:

$$\ln X_i(k) - \ln M = (\ln X_i^0(k) - \ln M) - \Sigma G_{i\alpha}\lambda_\alpha(k)$$

$$\ln (X_i(k)/M) \approx - \Sigma G_{i\alpha}\lambda_\alpha(k)$$

where M represents the median value.

Theoretically, the number of constraints that operate on the system is limited by the smaller dimension of the input matrix [7]. In our case it is limited by the number of macromolecules measured, i.e. in the current dataset, the levels of 181 proteins were measured, and therefore a maximum of 181 constraints, or unbalanced processes that operate on the system could have been found. However, most of these processes are insignificant, i.e. have a negligible weight, λ_α, in all samples. Our analysis revealed only 17 significant unbalanced processes, which reproduce the experimental expression levels of the proteins in the dataset (**Fig. S2**). Figure S4 demonstrates the robustness of surprisal analysis, and shows that the size of the dataset studied here is large enough. A smaller dataset, of 100 tumors of each type, did not change the results of our analysis.

Determination of the number of significant unbalanced processes. (see [15] for more details). The analysis of the 3467 patients provided a 3467x181 matrix of $\lambda_\alpha(k)$ values, such that every row in the matrices contained 3467 values of $\lambda_\alpha(k)$ for 3467 patients, and each row corresponded to an unbalanced process (**Table S2**). However, not all unbalanced processes are significant. Our goal is to determine how many unbalanced processes are needed in order to reconstruct the experimental data, i.e. for which value of *n*: ln $(X_i(k)/M) \approx - \Sigma G_{i\alpha}\lambda_\alpha(k)$. To find *n*, we performed the following two steps:

(1) **<u>Processes with significant amplitudes were selected</u>**: To calculate threshold limits for $\lambda_\alpha(k)$ values (presented in **Table S2** and **Fig. S5**) the standard deviations of the levels of 10 most stable proteins in this dataset were calculated (e.g. those with the smallest Standard deviations values, such as Annexin VII). Those fluctuations were considered as baseline fluctuations in the population of the patients which are not influenced by the unbalanced processes. Using

standard deviation values of these proteins the threshold limits were calculated as described previously [17]. The analysis revealed that from α = 18, the importance values, $\lambda_\alpha(k)$, become insignificant (i.e. do not exceed the noise threshold), suggesting that 17 unbalanced processes are enough to describe the system.

(2) **Reproduction of the experimental data by the unbalanced processes was verified**: To verify that the number of processes identified in step (1) is correct, we plotted $\Sigma G_{i\alpha}\lambda_\alpha(k)$ for $\alpha = 1, 2, \ldots, n$ against ln $X_i(k)$ for different proteins, i, and for different values of n, and examined the correlation between them as n was increased. An unbalanced process, $\alpha = n$, was considered significant if it improved the correlation significantly relative to $\alpha = n - 1$. Figure S2 exemplifies this process for pEGFR, showing that increasing n from 17 to 21 did not significantly affect the correlation.

Generation of functional subnetworks.

The functional subnetworks presented in Figure S1 were generated using a python script (written with the assistance of Mr. Jonathan Abramson). The goal was to generate a functional network according to STRING database, where proteins with negative G values are marked blue and proteins with positive G values are marked red, to easily identify the correlations and anti-correlations between the proteins in the network. The script takes as an input the names of the genes in the network and their G values, obtains the functional connections and their weights from STRING database (string-db.org), and then plots the functional network (using matplotlib library).

Note: Since the antibodies against pY(1248)ErbB2 and pY(1068)EGFR were noticed to cross react in the original RPPA assay[18], the following corrections were made to our analyses: In unbalanced processes in which both pY(1248)ErbB2 and pY(1068)EGFR were significant, EGFR was considered to be active only if pY(1173)EGFR was significant as well. pY(1248)ErbB2 was considered to be a false-positive result and was thus omitted from these processes. Therefore, pY(1248)ErbB2 was omitted from the unbalanced processes $\alpha = 1,5,10,13,14$.

Calculation of barcodes.

The barcodes presented in Table S4, Figures 4-7 and Figure S6 were generated using a python script (written with the assistance of Mr. Jonathan Abramson). For each patient, $\lambda_\alpha(k)$ (α = 1, 2, 3, …, 17) values were normalized as follows: If $\lambda_\alpha(k) > 2$ (and is therefore significant according to calculation of threshold values) then is was normalized to 1; if $\lambda_\alpha(k) < -2$ (significant according to threshold values as well) then it was normalized to -1; and if $-2 < \lambda_\alpha(k) < 2$ then it

was normalized to 0. Table S4 lists the 452 unique barcodes that were identified, their frequency in each cancer type and their overall frequency in all 3467 tumors. The results are shown graphically in Figures 4-7 and in Figure S6.

Cell lines and reagents.

MDA-MB-231 (triple negative breast cancer), MDA-MB-468 (triple negative breast cancer), and MCF7 (lumA breast cancer) were obtained from the American Type Culture Collection (ATCC) and cultured in RPMI with 10% fetal calf serum (FCS), supplemented with 100 U/ml penicillin and 100 mg/ml streptomycin, and grown at 37ºC/5% CO2. The cell lines were authenticated at the Biomedical Core Facility of the Technion, Haifa, Israel.

Methylene blue (sc-215381) and anti-T(202)Y(204)ERK^{MAPK} (sc-7383) were from Santa Cruz Biotechnology (Santa Cruz, CA), 4-hydroxy-Tamoxifen (H7904) was from Sigma Aldrich (St. Louis, MO), anti-pY(1068)EGFR (#3777), anti-PARP (#9542), anti-pS(235/236)S6 (#4858) were from Cell Signaling Technology (Beverly, MA), 2-deoxy-D-Glucose was from MP Biochemicals (Santa Ana, CA), HRP-conjugated goat anti-mouse and HRP-conjugated goat anti-rabbit were from Jackson ImmunoResearch Laboratories, Inc. (West Grove, PA), trametinib, erlotinib and LY2584702 were from Cayman Chemicals (Ann Arbor, MI), taxol (paclitaxel) was from Teva Pharmaceutical Industries, Ltd. (Israel).

Methylene blue assays.

Cells were seeded in 96-well plates and grown under complete medium. The next day, inhibitors were added for 72 hours. The cells were then fixed and the amount of surviving cells was quantified by staining with methylene blue (Sigma) as described in[19].

Western blot analysis.

Cells were seeded in 6-well plates and grown under complete medium. The next day, cells were treated as indicated for 48 hours, and then the cells were collected into clean tubes (including the fraction of floating dead cells) and lysed using hot sample buffer (10% glycerol, 50 mmol/L Tris-HCl pH 6.8, 2% SDS, and 5% 2-mercaptoethanol). Western blot analysis was carried out as described previously [20].

Supplementary Figures and legends

Figure S1.

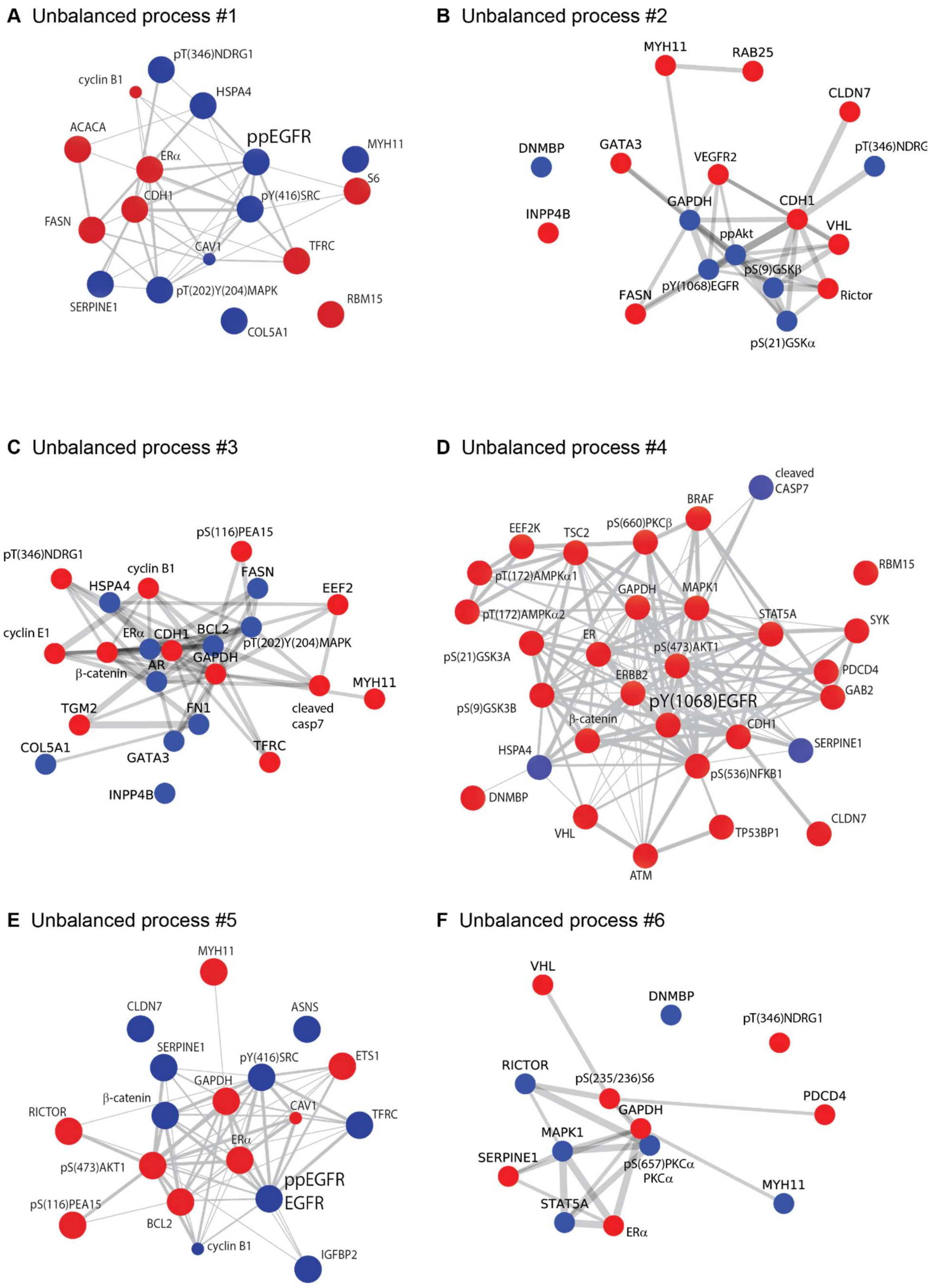

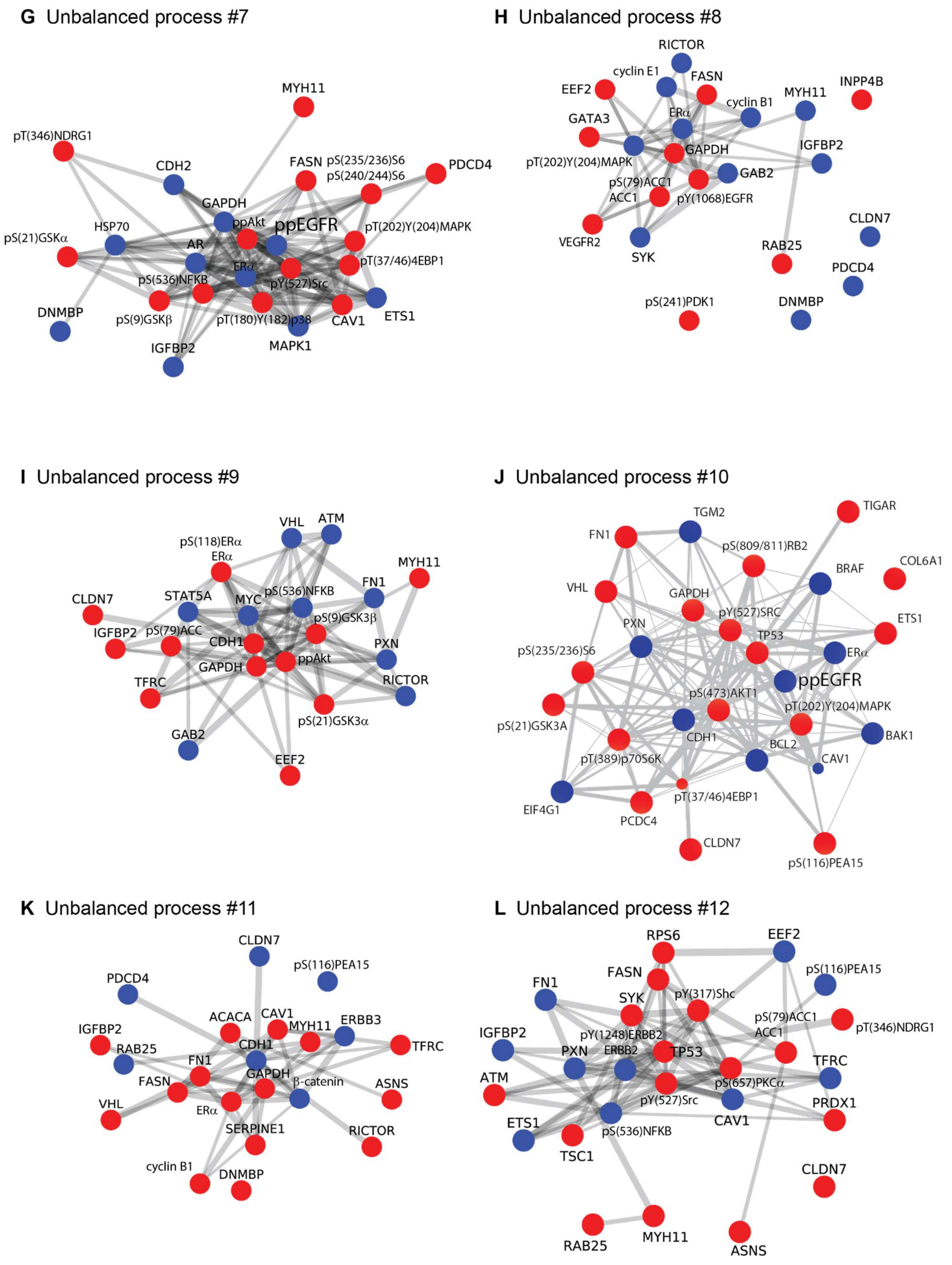
G Unbalanced process #7
MYH11
pT(346)NDRG1
CDH2
FASN
pS(235/236)S6
pS(240/244)S6
PDCD4
GAPDH
ppAkt
ppEGFR
pT(202)Y(204)MAPK
HSP70
pS(21)GSKα
AR
ERα
pT(37/46)4EBP1
pS(536)NFKB
pY(527)Src
DNMBP
pS(9)GSKβ
pT(180)Y(182)p38
CAV1
ETS1
IGFBP2
MAPK1
H Unbalanced process #8
RICTOR
cyclin E1
FASN
EEF2
cyclin B1
MYH11
INPP4B
GATA3
ERα
GAPDH
IGFBP2
pT(202)Y(204)MAPK
GAB2
pS(79)ACC1
ACC1
pY(1068)EGFR
VEGFR2
SYK
CLDN7
RAB25
PDCD4
pS(241)PDK1
DNMBP
I Unbalanced process #9
VHL
ATM
pS(118)ERα
ERα
MYH11
CLDN7
STAT5A
MYC
pS(536)NFKB
FN1
pS(9)GSK3β
IGFBP2
pS(79)ACC
CDH1
PXN
GAPDH
ppAkt
TFRC
RICTOR
pS(21)GSK3α
GAB2
EEF2
J Unbalanced process #10
TGM2
TIGAR
FN1
pS(809/811)RB2
COL6A1
BRAF
VHL
GAPDH
pY(527)SRC
ETS1
PXN
TP53
pS(235/236)S6
ERα
ppEGFR
pS(473)AKT1
pT(202)Y(204)MAPK
pS(21)GSK3A
CDH1
BAK1
BCL2
pT(389)p70S6K
CAV1
pT(37/46)4EBP1
EIF4G1
PCDC4
CLDN7
pS(116)PEA15
K Unbalanced process #11
CLDN7
pS(116)PEA15
PDCD4
ACACA
CAV1
MYH11
ERBB3
IGFBP2
CDH1
TFRC
RAB25
FN1
GAPDH
β-catenin
FASN
ASNS
VHL
ERα
SERPINE1
RICTOR
cyclin B1
DNMBP
L Unbalanced process #12
RPS6
EEF2
FASN
pS(116)PEA15
FN1
SYK
pY(317)Shc
pS(79)ACC1
ACC1
pT(346)NDRG1
IGFBP2
pY(1248)ERBB2
ERBB2
PXN
TP53
TFRC
ATM
pS(657)PKCα
pY(527)Src
CAV1
PRDX1
ETS1
pS(536)NFKB
TSC1
CLDN7
RAB25
MYH11
ASNS

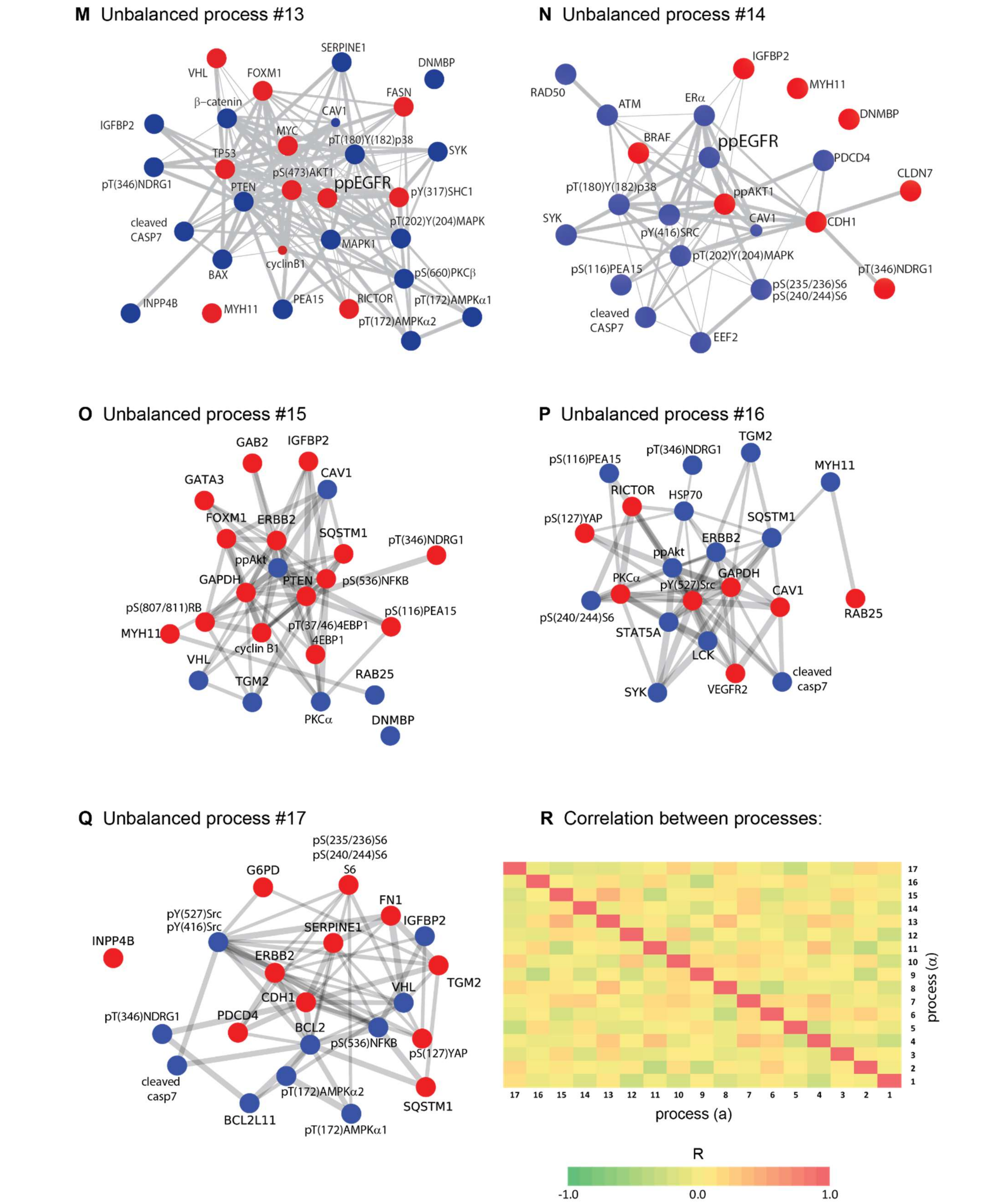

Figure.S1 The unbalanced subnetworks identified by surprisal analysis for 3467 tumors. (A-Q) For every process α, the proteins were assembled into networks using functional interactions according to STRING database. ppAkt = Akt phosphorylated on Thr308 and on Ser473; ppEGFR = EGFR phosphorylated on Tyr1068 and on Tyr1173. (**R**) The correlation index, R, was calculated for every pair of processes. The results are shown in heatmap form and demonstrate the independence of the 17 processes.

Figure S2.

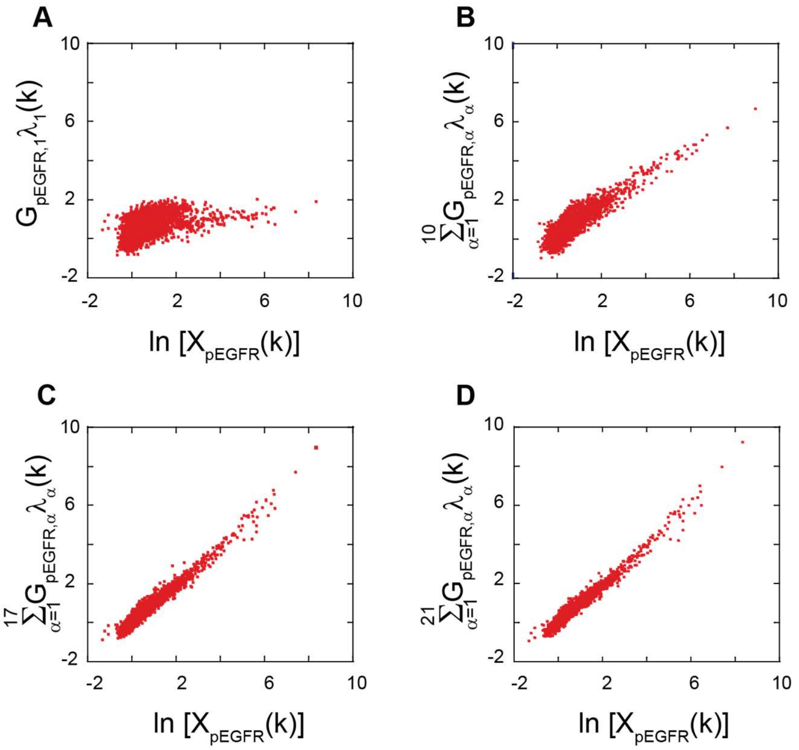

Figure. S2 17 unbalanced processes span the entire biological imbalance in 3467 tumors. To show that the first 17 unbalanced processes are enough to reproduce the dataset, we looked whether the changes in the experimental protein expression levels can be fitted well by 17 unbalanced processes that appeared to be significant (exceed the threshold limits). For example, for every tumor k, the theoretical expression level of pEGFR in this tumor ($\Sigma G_{pEGFR,\alpha}\lambda_{\alpha}(k)$ for $\alpha = 1, 2, \ldots, n$) was plotted against the experimental expression level ($\ln X_{pEGFR}(k)$), in 4 different cases: (**A**) Only the first, most significant unbalanced process was taken into account (n=1). (**B**) The 10 most significant unbalanced processes were taken into account (n=10); (**C**) The 17 most significant unbalanced processes were taken into account (n=17); (**D**) The 21 most significant unbalanced processes were taken into account (n=21). Theoretically, if the experimental values exactly coincide with the theoretical values, all of the points on the graph should fall on the y=x line. The plots nicely shows how 17 unbalanced processes are enough to reproduce the expression levels of EGFR in all tumors.

Importantly, processes α=16 and α=17 had significant $\lambda_{\alpha}(k)$ values ($\lambda_{\alpha}(k) > 2$ or $\lambda_{\alpha}(k) < -2$) in some of the patients (see. For example, **Fig. S5**) and thus had to be considered. In contrast, processes α = 18, 19, … , 181 did not have significant values in any of the patients. Moreover, the plot of the data reproduction did not change significantly when the processes α = 18, 19, 20, 21 were added (see panel **C** vs. panel **D**). Thus, we conclude that the current dataset is well characterized by 17 unbalanced processes.

Figure S3.

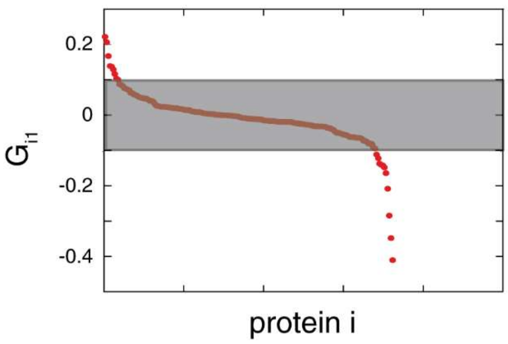

Figure. S3 Only proteins with significant $G_{i\alpha}$ values are considered to participate in the unbalanced process α. The proteins that take part in the different unbalanced processes were identified as follows: For every unbalanced process α, $G_{i\alpha}$ values were sorted according to their size, and only proteins with significant $G_{i\alpha}$ values were considered to participate in the unbalanced process α. This is exemplified for the process α = 1 in the figure. Shown are sorted values of G_{i1}, which represent the degree of participation of every protein i in the unbalanced process α = 1. The gray box represents threshold values. Proteins with $G_{i1} > 0.1$ or $G_{i1} < -0.1$ (which are not contained in the gray box and form the top and bottom "tails" of the distribution) were considered to participate the most in the unbalanced process α = 1. These proteins were used to build a functional subnetwork using STRING database, presented in Figure S1.

Figure S4.

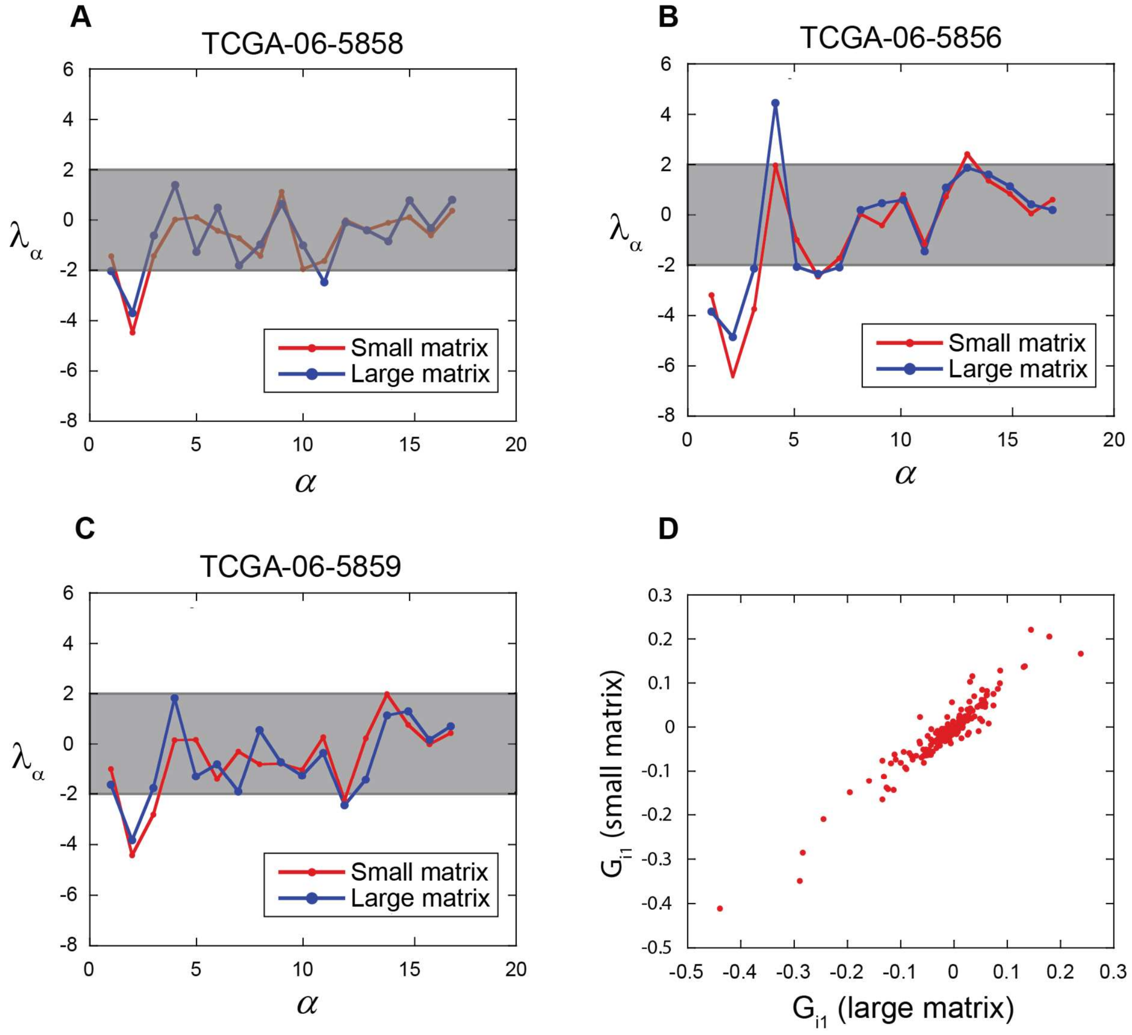

Figure. S4 Patient diagnoses remain essentially the same when a smaller matrix was analyzed. Since our technique involves identifying patient-specific unbalanced processes based on studying a large number of tumors at once, it is relevant to ask whether the size of the dataset analyzed here is large enough to be considered a representative sample. To address this, 100 patients from each tumor type were randomly chosen, and the same analysis was performed on this smaller matrix. The results are presented in the figure. **(A-C)** λ_α values for 3 random patients are shown, demonstrating that their diagnoses remain essentially the same when only 1100 tumors were used for the analysis instead of 3467. **(D)** G_{i1} values obtained from the analysis of the small matrix were plotted against G_{i1} values obtained from the analysis of the original matrix, demonstrating that the results are very similar.

Figure S5.

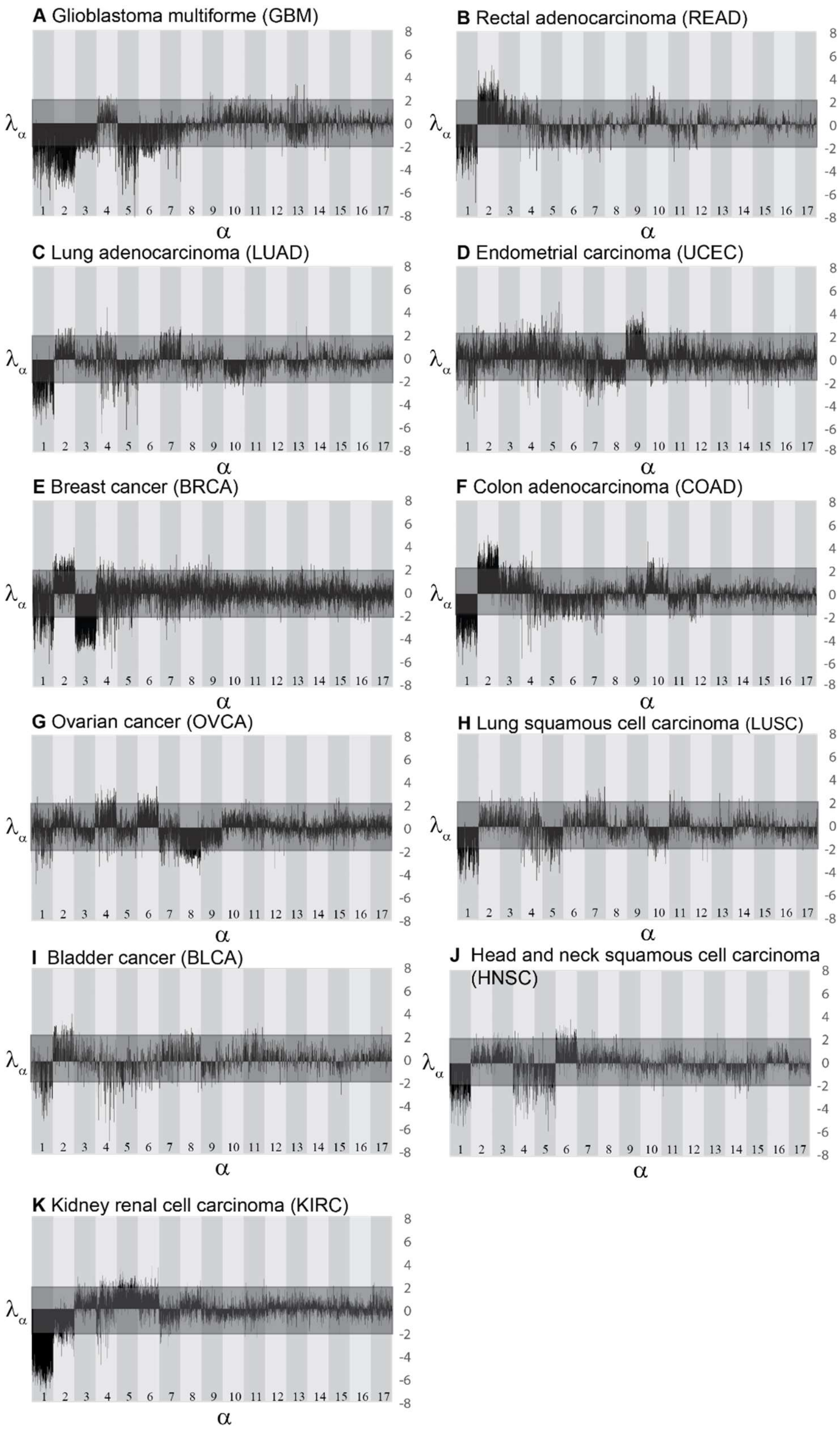

Figure S5. While some unbalanced processes frequently characterize specific tumor types, no tumor type carries a specific signature. The figure presents $\lambda_\alpha(k)$ values of the 17 unbalanced processes for all tumor types. The gray boxes mark threshold limits.

Figure S6.

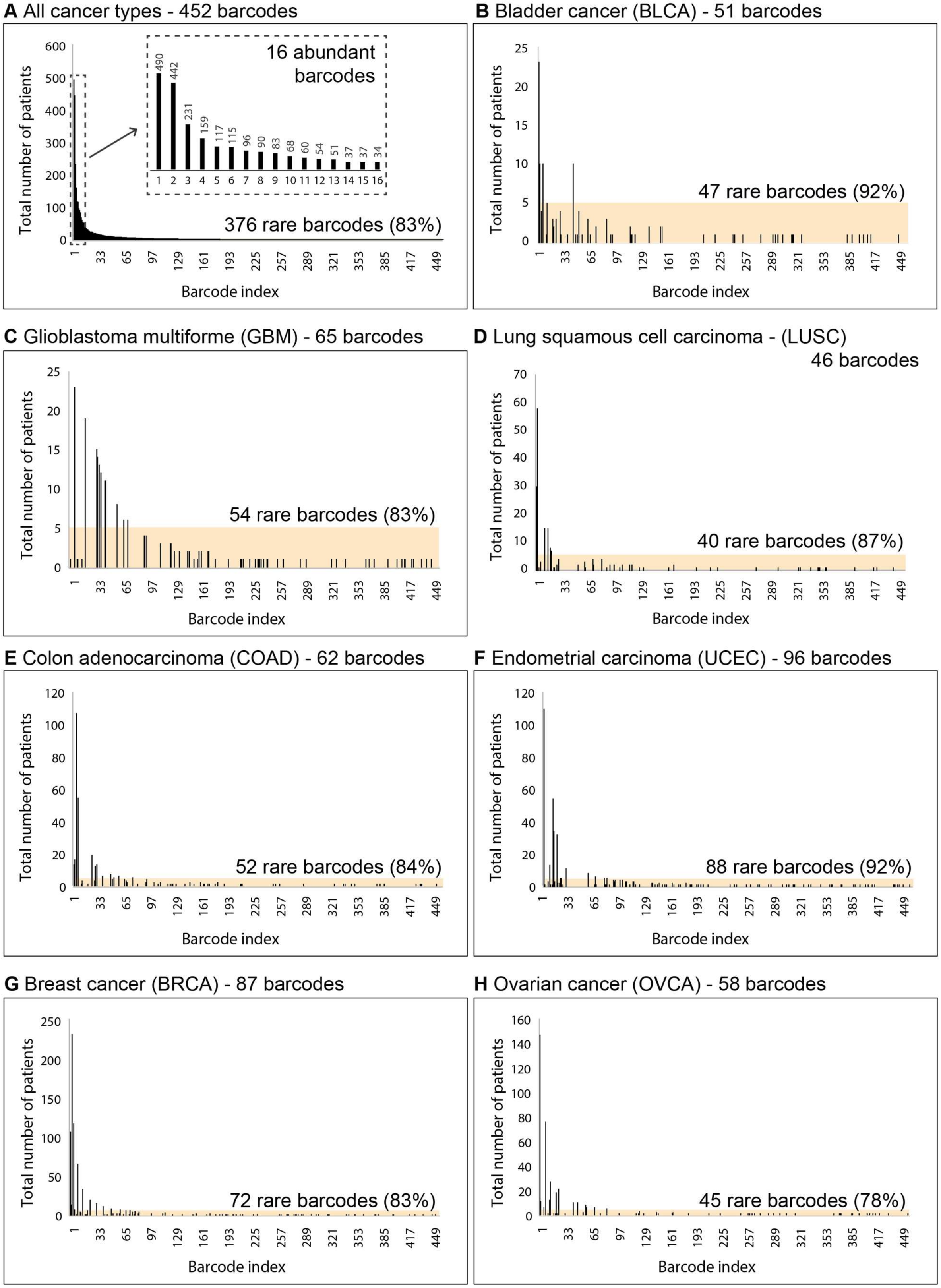

A All cancer types - 452 barcodes
16 abundant barcodes
490
442
231
159
117
115
96
90
83
68
60
54
51
37
37
34
376 rare barcodes (83%)
B Bladder cancer (BLCA) - 51 barcodes
47 rare barcodes (92%)
C Glioblastoma multiforme (GBM) - 65 barcodes
54 rare barcodes (83%)
D Lung squamous cell carcinoma - (LUSC) 46 barcodes
40 rare barcodes (87%)
E Colon adenocarcinoma (COAD) - 62 barcodes
52 rare barcodes (84%)
F Endometrial carcinoma (UCEC) - 96 barcodes
88 rare barcodes (92%)
G Breast cancer (BRCA) - 87 barcodes
72 rare barcodes (83%)
H Ovarian cancer (OVCA) - 58 barcodes
45 rare barcodes (78%)
Total number of patients
Barcode index

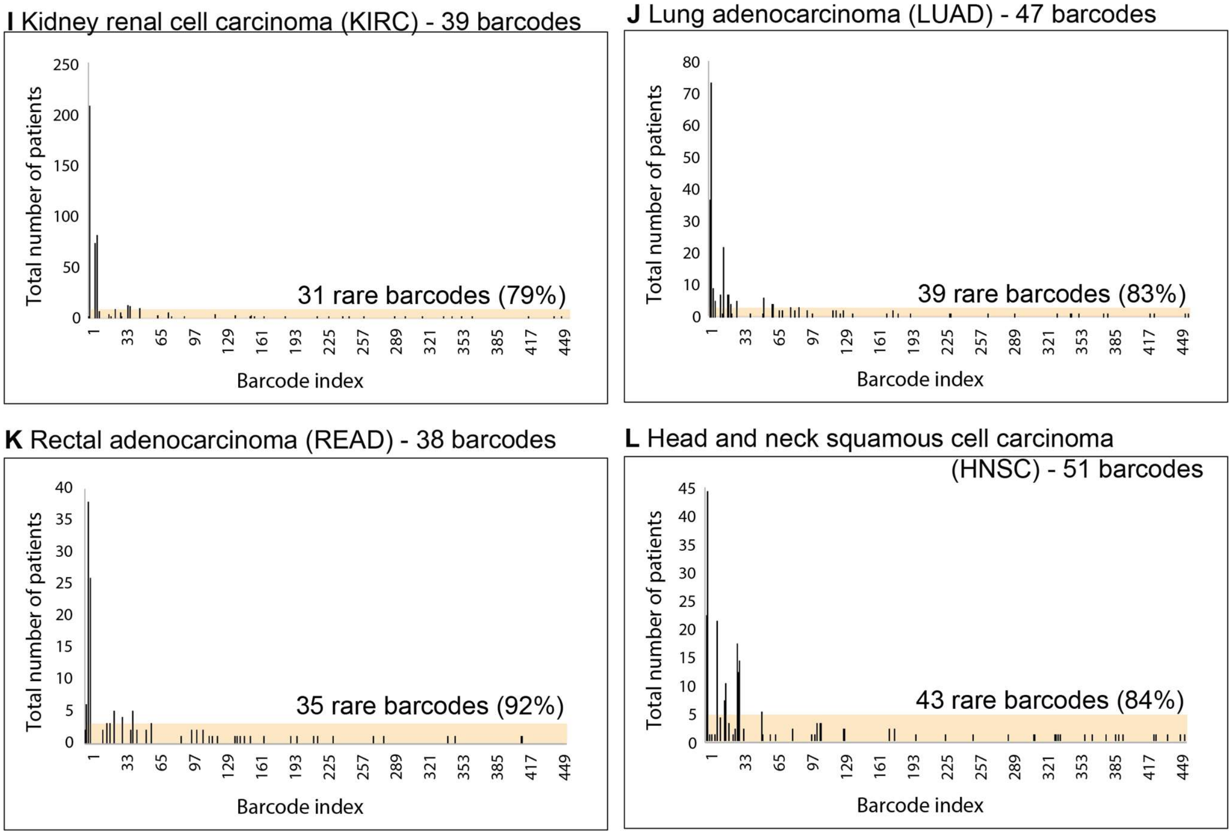

Figure S6. The majority of tumors are characterized by rare barcodes. (**A**) *452 unique barcodes characterize the entire population of 3467 tumors.* The different barcodes were sorted according to their frequency in the entire population of tumors. The dashed box shows the 16 most abundant barcodes. The rare barcodes (376 barcodes which characterize only 5 tumors or less) are highlighted in orange. (**B-L**) *The majority of tumor type-specific barcodes are rare.* For each of the tumor types, the collection of unique barcodes that characterize the specific tumor type were plotted. Rare barcodes are highlighted in orange.

Figure S7.

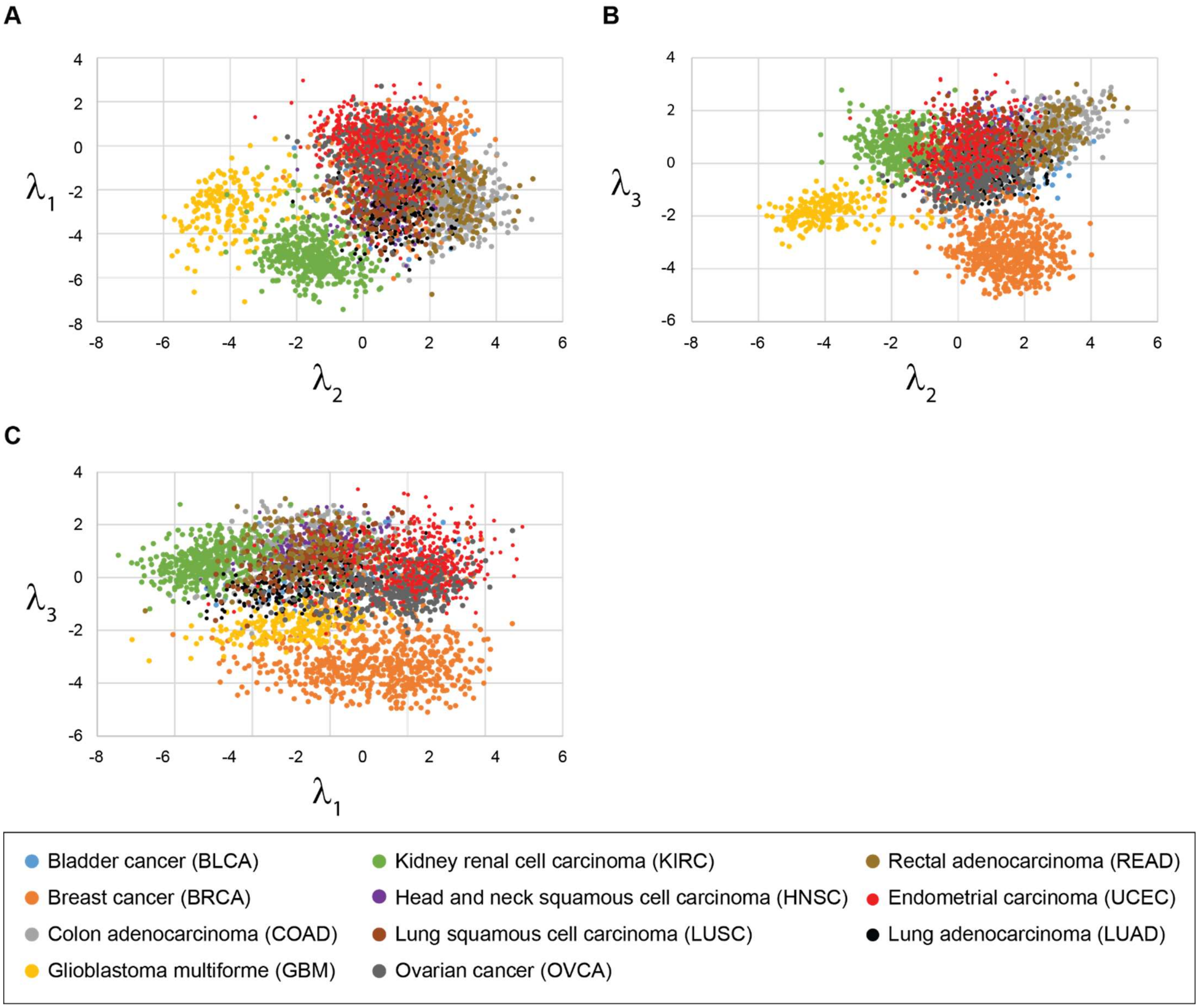

Figure S7. A two-dimensional representation of the tumor imbalances masks the patient-explicit network structure. (**A-C**) The amplitudes of the three most significant unbalanced processes, $\lambda_1(k)$, $\lambda_2(k)$, and $\lambda_3(k)$, were plotted against each other in pairs in order to examine the information that these plots can provide. Some cancer types are nicely separated, e.g. GBM, KIRC and BRCA by λ_2 (panels a and b) and λ_3 (panels b and c). However, though some cancer types can be separated to some extent according to these 3 processes, representation of tumors using these 2D graphs does not enable the accurate mapping of individual tumors. Many tumors with distinct protein network structures appear in the same place in these graphs, e.g. most UCEC and HNSC tumors, which we found to be highly heterogeneous. These results demonstrate that a partial identification of unbalanced molecular processes does not suffice to accurately map the cancer patients.

Figure S8.

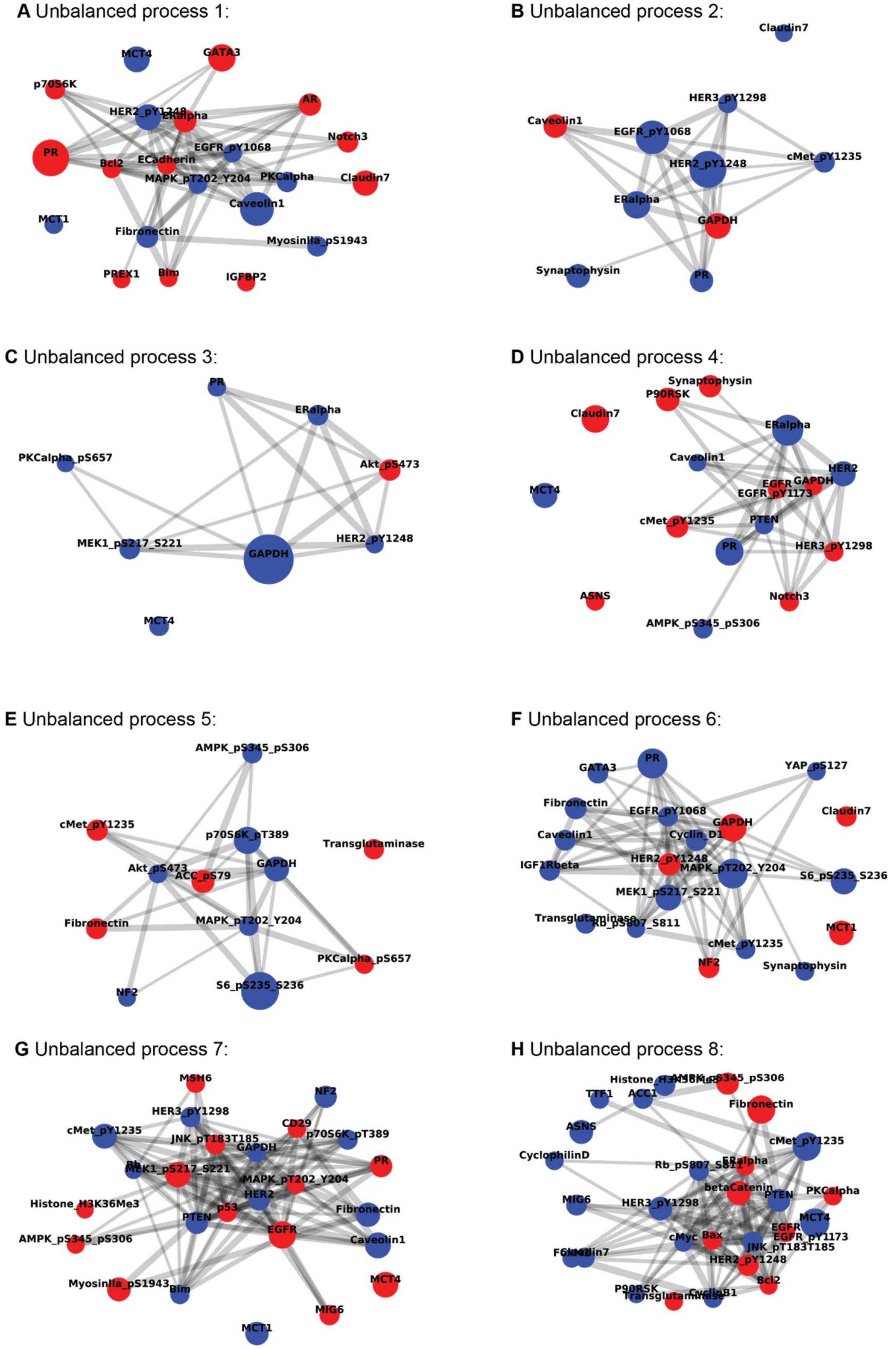

Figure S8. The unbalanced subnetworks identified by surprisal analysis for 10 cell lines. For every process α, the proteins were assembled into networks using functional interactions according to STRING database.

Figure S9.

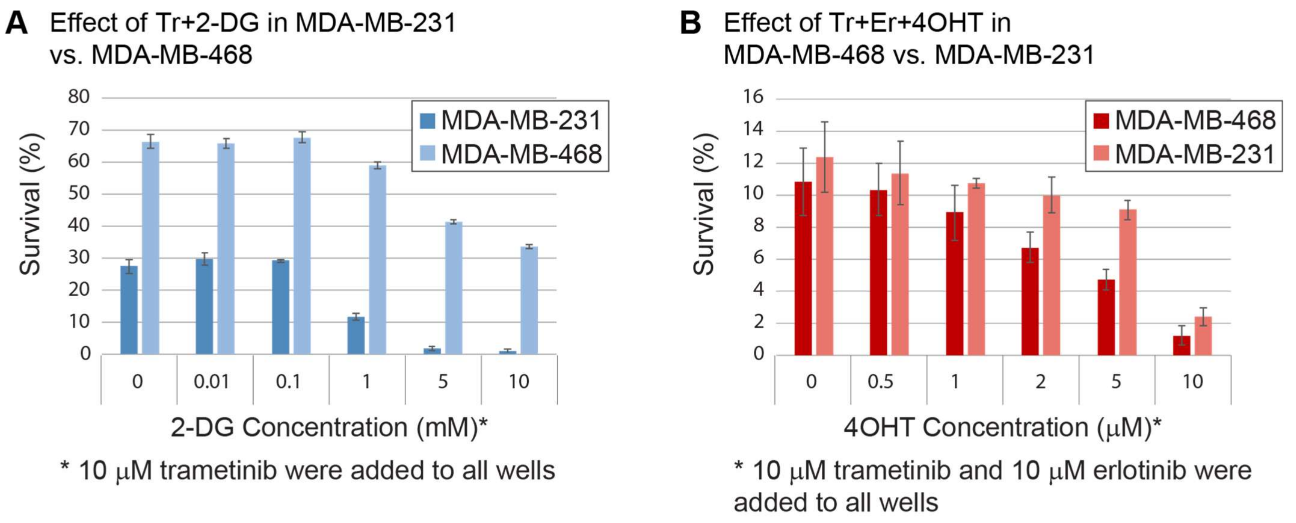

Figure S9. Different combinations of drugs are effective against different TNBC cell lines. Trametinib + 2-DG was the combination of drugs predicted for MDA-MB-231. We compared the effect of this combination on the survival of MDA-MB-231 to its effect on the survival of MDA-MB-468 (**A**). Similarly, the effect of the combination predicted for MDA-MB-468, trametinib + erlotinib + 4OHT, on the survival of MDA-MB-468 was compared to its effect on the survival of MDA-MB-231 (**B**). The results shown demonstrate that the predicted combinations were cell line-specific, and suggest that different TNBC tumors may benefit from different combinations of drugs, that are specifically tailored to their altered signaling signature.

Figure S10.

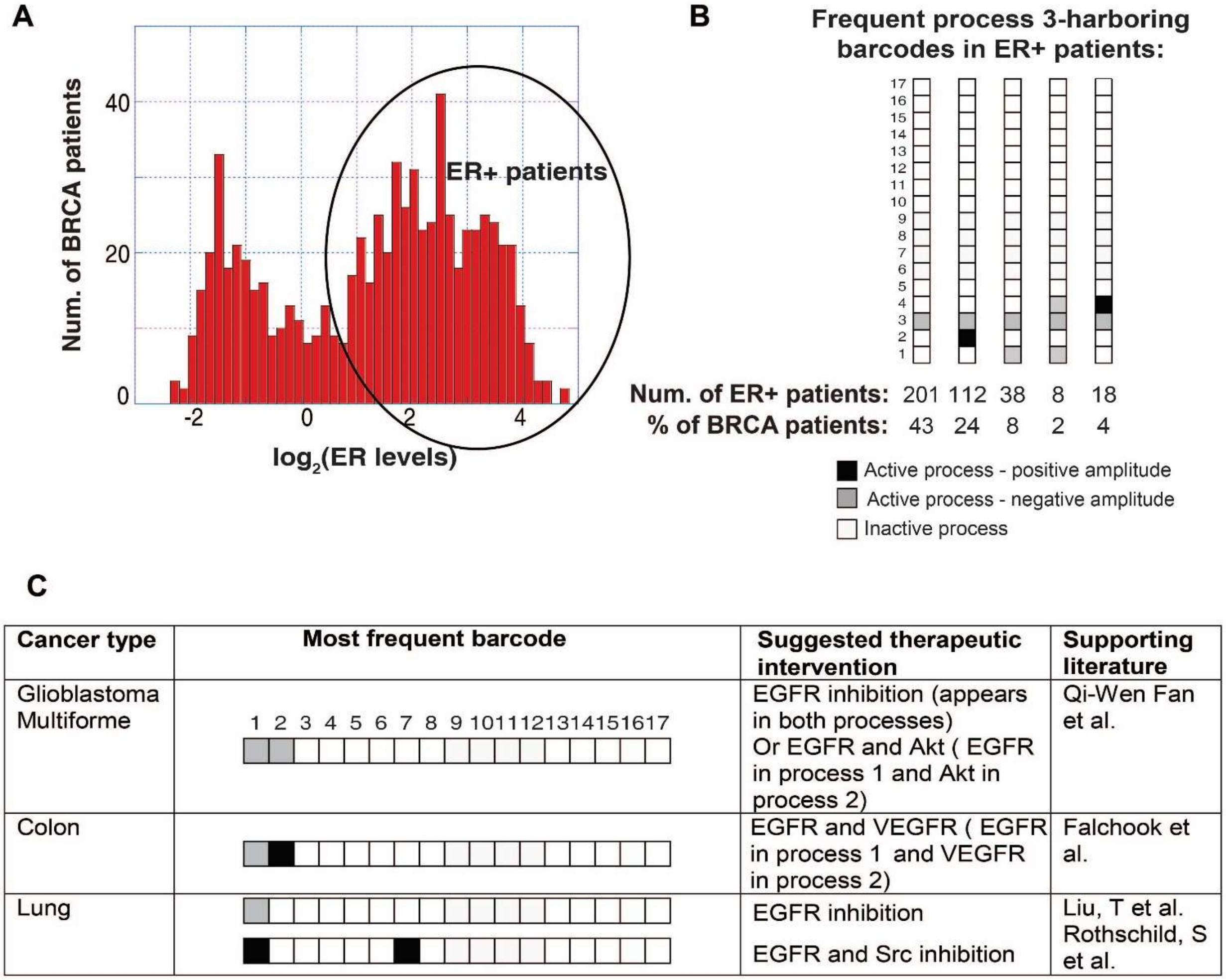

Figure S10. Literature support for the combined therapies suggested by the analysis. (A-B) The most frequent barcodes in 469 *ER positive* breast cancer patients (ER positive were defined as breast cancer patients in the dataset with log2(ER expression levels) > 1, see encircled subpopulation in (A)) are shown (B). 43% of 469 ER positive patients, have a barcode with active process 3 (B) and would be suggested to receive an anti-ER monotherapy, corresponding to the current clinical routine. However, other patients (4 additional barcodes are shown for ER positive subgroups comprising > 1% of ER positive subpopulation (B)) would benefit from combined therapies that include Ramucirumab + Tamoxifen or Erlotinib + Tamoxifen (Table S7). These combinations are proposed by the literature as potential effective combinations for ER resistant breast cancer ([21,22]. (C) Most frequent barcodes are shown for Glioblastoma Multiforme (~11% of GBM patients), Colon (~32% of colon cancer patients) and Lung adenocarcinoma (31% and 9% of lung cancer patients, Table S4). The suggested therapies for these barcodes (Table S8) are proposed by the literature as potential effective combinations for those cancer types [23–26].

Figure S11.

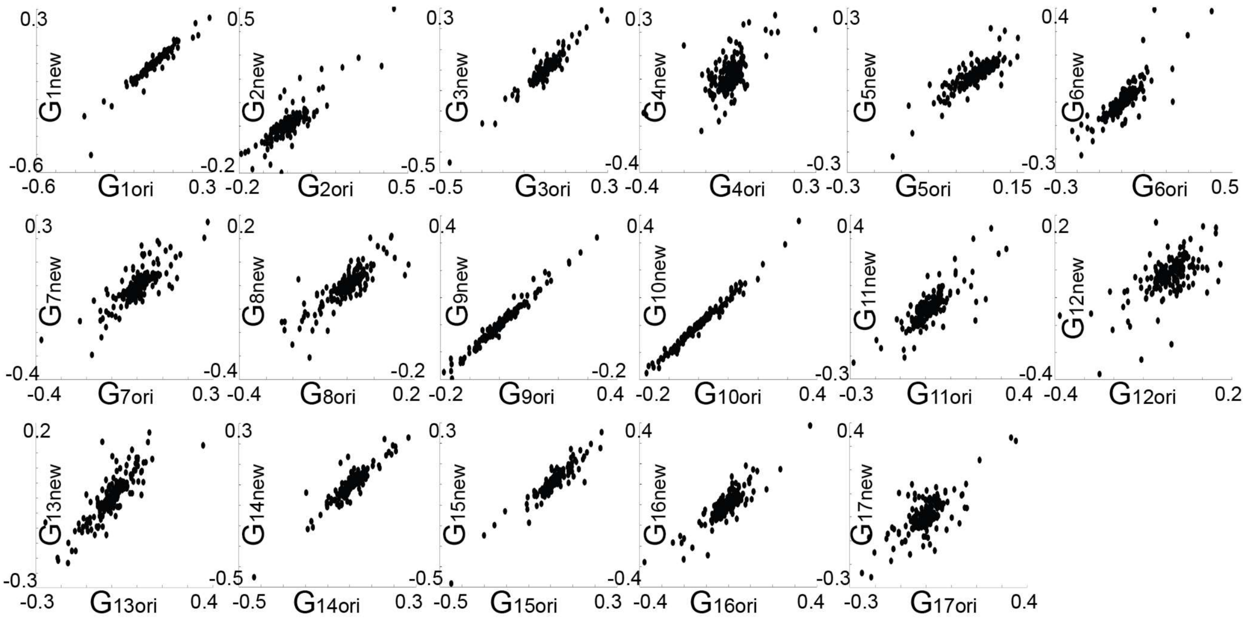

Figure S11. Protein composition of unbalanced processes remained the same before and after addition of the prostate carcinoma dataset. The weights of the proteins in every unbalanced process from the first dataset were highly correlated with the weights of the proteins after addition of the prostate carcinoma dataset. The correlation coefficient R is above 0.7 for 15 processes, meaning that the correlation is strong, for only 2 processes R was above 0.5-0.6, pointing to moderately strong correlation. Ori -denotes analysis of the original dataset and new- denotes the values after adding the validation (prostate cancer) dataset.

References

1. Levine RD, Bernstein RB. Energy disposal and energy consumption in elementary chemical reactions. Information theoretic approach. Acc Chem Res. 1974;7(12):393–400.

2. Levine RD. An information theoretical approach to inversion problems. J Phys A Math Gen. 1980;13(1):91.

3. Levine RD. Molecular Reaction Dynamics [Internet]. Cambridge: The University Press. Cambridge: The University Press; 2005.

4. McMillan WG, Mayer JE. THE STATISTICAL THERMODYNAMICS OF MULTICOMPONENT SYSTEMS. J Chem Phys. 1945;13(7):276–305.

5. Mayer JE, Mayer MG. Statistical mechanics. 2nd ed. New York: Wiley; 1977.

6. McQuarrie DA. Statistical Mechanics, 1st ed. [Internet]. University science books. 2000.

7. Remacle F, Kravchenko-Balasha N, Levitzki A, Levine RD. Information-theoretic analysis of phenotype changes in early stages of carcinogenesis. Proc Natl Acad Sci U S A. 2010;107(22):10324–9.

8. Kravchenko-Balasha N, Levitzki A, Goldstein A, Rotter V, Gross A, Remacle F, et al. On a fundamental structure of gene networks in living cells. Proc Natl Acad Sci U S A. 2012/03/07. 2012;109(12):4702–7.

9. Kravchenko-Balasha N, H. Johnson, F.M. White, J.R. Heath, Levine R.D. A Thermodynamic Based Interpretation of Protein Expression Heterogeneity in Different GBM Tumors Identifies Tumor Specific Unbalanced Processes. J Phys Chem B. 2016;120(26):5990–7.

10. Zadran S, Remacle F, Levine RD. miRNA and mRNA cancer signatures determined by analysis of expression levels in large cohorts of patients. Proc Natl Acad Sci U S A. 2013;110(47):19160–5.

11. David L. Nelson, Albert L. Lehninger , Cox MM. Lehninger Principles of Biochemistry. 5th ed. Macmillan; 2008.

12. Kravchenko-Balasha N, Wang J, Remacle F, Levine RD, Heath JR. Glioblastoma cellular architectures are predicted through the characterization of two-cell interactions. Proc Natl Acad Sci U S A. 2014;111(17):6521–6.

13. Kravchenko-Balasha N, Shin YS, Levine R. D., Heath JR. Intercellular Signaling Through Secreted Proteins Induces Free Energy Gradient-directed Cell Movement. Proc Natl Acad Sci U S A. 2016;(accepted).

14. Flashner-Abramson E, Abramson J, White FM, Kravchenko-Balasha N. A thermodynamic-based approach for the resolution and prediction of protein network structures. Chem Phys. 2018;514:20–30.

15. Vasudevan S, Flashner-Abramson E, Remacle F, Levine RD, Kravchenko-Balasha N. Personalized disease signatures through information-theoretic compaction of big cancer data. Proc Natl Acad Sci U S A. 2018;115(30):7694–9.

16. Kravchenko-Balasha N, Johnson H, White FM, Heath JR, Levine RD. A Thermodynamic-Based Interpretation of Protein Expression Heterogeneity in Different Glioblastoma Multiforme Tumors Identifies Tumor-Specific Unbalanced Processes. J Phys Chem B. 2016;

17. Gross A, Levine RD. Surprisal analysis of transcripts expression levels in the presence of noise: a reliable determination of the onset of a tumor phenotype. PLoS One. 2013/04/30. 2013;8(4):e61554.

18. Akbani R, Ng PKS, Werner HMJ, Shahmoradgoli M, Zhang F, Ju Z, et al. A pan-cancer proteomic perspective on The Cancer Genome Atlas. Nat Commun. 2014;5:3887.

19. Zenvirt S, Kravchenko-Balasha N, Levitzki A. Status of p53 in human cancer cells does not predict efficacy of CHK1 kinase inhibitors combined with chemotherapeutic agents. Oncogene. 2010;29(46):6149–59.

20. Reuveni H, Flashner-Abramson E, Steiner L, Makedonski K, Song R, Shir A, et al. Therapeutic destruction of insulin receptor substrates for cancer treatment. Cancer Res. 2013;73(14):4383–94.

21. Rugo HS, Vidula N, Ma C. Improving Response to Hormone Therapy in Breast Cancer: New Targets, New Therapeutic Options. Am Soc Clin Oncol Educ B. 2016;(36):e40–54.

22. Patel RR, Sengupta S, Kim HR, Klein-Szanto AJ, Pyle JR, Li T, et al. Experimental treatment of oestrogen receptor (ER) positive breast cancer with tamoxifen and brivanib alaninate, a VEGFR-2/FGFR-1 kinase inhibitor: A potential clinical application of angiogenesis inhibitors. Eur J Cancer. 2010;46:1537–53.

23. Fan QW, Cheng C, Gustafson WC, Charron E, Zipper P, Wong R, et al. EGFR Phosphorylates Tumor-Derived EGFRvIII Driving STAT3/5 and Progression in Glioblastoma. Cancer Cell. 2013;24(4):438–49.

24. Falchook GS, Kurzrock R. VEGF and dual-EGFR inhibition in colorectal cancer. Cell Cycle. 2015;14(8):1129.

25. Liu F, Mischel PS, Cavenee WK. Precision cancer therapy is impacted by oncogene-dependent epigenome remodeling. npj Precis Oncol. 2017;1(1):1.

26. Rothschild SI, Gautschi O. Src tyrosine kinase inhibitors in the treatment of lung cancer: rationale and clinical data. Clin Investig (Lond). 2012;
